# Evaluation of shotgun metagenomics sequence classification methods using *in silico* and *in vitro* simulated communities

**DOI:** 10.1186/s12859-015-0788-5

**Published:** 2015-11-04

**Authors:** Michael A. Peabody, Thea Van Rossum, Raymond Lo, Fiona S. L. Brinkman

**Affiliations:** Department of Molecular Biology and Biochemistry, Simon Fraser University, Burnaby, BC Canada

**Keywords:** Metagenomics, Evaluation, Accuracy, Comparison, Taxonomic classification

## Abstract

**Background:**

The field of metagenomics (study of genetic material recovered directly from an environment) has grown rapidly, with many bioinformatics analysis methods being developed. To ensure appropriate use of such methods, robust comparative evaluation of their accuracy and features is needed. For taxonomic classification of sequence reads, such evaluation should include use of clade exclusion, which better evaluates a method’s accuracy when identical sequences are not present in any reference database, as is common in metagenomic analysis. To date, relatively small evaluations have been performed, with evaluation approaches like clade exclusion limited to assessment of new methods by the authors of the given method. What is needed is a rigorous, independent comparison between multiple major methods, using the same *in silico* and *in vitro* test datasets, with and without approaches like clade exclusion, to better characterize accuracy under different conditions.

**Results:**

An overview of the features of 38 bioinformatics methods is provided, evaluating accuracy with a focus on 11 programs that have reference databases that can be modified and therefore most robustly evaluated with clade exclusion. Taxonomic classification of sequence reads was evaluated using both *in silico* and *in vitro* mock bacterial communities. Clade exclusion was used at taxonomic levels from species to class—identifying how well methods perform in progressively more difficult scenarios. A wide range of variability was found in the sensitivity, precision, overall accuracy, and computational demand for the programs evaluated. In experiments where distilled water was spiked with only 11 bacterial species, frequently dozens to hundreds of species were falsely predicted by the most popular programs. The different features of each method (forces predictions or not, etc.) are summarized, and additional analysis considerations discussed.

**Conclusions:**

The accuracy of shotgun metagenomics classification methods varies widely. No one program clearly outperformed others in all evaluation scenarios; rather, the results illustrate the strengths of different methods for different purposes. Researchers must appreciate method differences, choosing the program best suited for their particular analysis to avoid very misleading results. Use of standardized datasets for method comparisons is encouraged, as is use of mock microbial community controls suitable for a particular metagenomic analysis.

**Electronic supplementary material:**

The online version of this article (doi:10.1186/s12859-015-0788-5) contains supplementary material, which is available to authorized users.

## Background

Metagenomics involves collecting samples from an environment (water, saliva, etc.) and then extracting and studying the genetic material from the microorganisms present in these samples [1]. This approach is transforming microbiology, ecology, medicine, and other research areas investigating various microbiomes, allowing us to analyze for the first time microbial species, including those not culturable, at a level of detail not previously possible [2]. Metagenomics sequence reads can be taxonomically classified to identify the microbes, or functionally classified (gene functions, metabolic pathways, etc.) to identify the functional potential of the community. There exist two general approaches for characterizing the taxonomic content of environmental samples: (1) sequencing of PCR amplicons corresponding to phylogenetic marker genes (e.g. 16S rRNA; “amplicon analysis”); (2) shotgun sequencing whereby all genomic DNA in the community is sequenced. A drawback of the shotgun sequencing approach is increased cost, but advantages include the ability to gain insights into metabolism and gene function through functional classification, and the avoidance of potentially biased amplification steps [3]. Furthermore, a notable subset of taxa cannot be captured by traditional 16S sequencing owing to divergent 16S rRNA gene sequences [4]. This, combined with the continuing decrease in cost of sequencing, may result in shotgun metagenomics becoming increasingly used for the taxonomic classification of microbial communities.

Taxonomic classification methods generally fall into four categories, reflecting their different strategies: (1) sequence similarity based methods, which use the results of a sequence similarity search against a database of a reference set of sequences, (2) sequence composition based methods, which are based on characteristics of their nucleotide composition (e.g. tetranucleotide usage or codon usage) [5], (3) hybrid methods which incorporate components of the first two, and (4) marker-based methods which identify species based on the occurrence of certain specific marker sequences. Composition methods generate models from the reference organisms’ genomes, and will classify the input sequence reads based on which model(s) fit the read best. They have had trouble with classifying reads of short length (<1000 base pairs), with Phymm being the first method published demonstrating reasonable accuracy at short read lengths [6]. Sequence similarity based methods, on the other hand, perform very well at identifying reads from genomes within the reference database that they search against, even at read lengths as short as 80 base pairs [7]. However, many reads from metagenomics samples come from genomes that are not in any reference database [8]. Similarity based methods have traditionally used BLAST [9], and have been generally slower to run compared to composition based methods. Hybrid methods combine the similarity approach and the composition approach, with the goal of improving classification or speed. For improving classification, scores may be combined from both the similarity portion and the composition portion of the method for each prediction [6]. Another hybrid strategy, aimed at increasing speed, is to use the composition approach to narrow down the set of candidate organisms, and thus have the similarity search occur against a fraction of the original database [10].

A related group of methods try to determine community composition from metagenomes by utilizing marker genes. These methods differ from methods that perform taxonomic classification, as they do not to try to classify all of the reads. Instead, they focus on classifying only marker genes to try to determine the microbial community composition of the sample. Most marker based approaches utilize universal genes. However, another approach, utilized by MetaPhlAn, involves use of clade-specific marker genes [11].

The first step in a marker based approach is to identify reads that hit to one of the markers. As the size of the reference database of markers these methods use is relatively small, these methods are comparatively quick to run. In addition to focusing on a limited set of markers, which greatly reduce the computational cost of analysis, these methods are not affected by differences in genome size. If the goal of the analysis is to identify the community composition of the sample, taxonomic classification methods are biased by genome sizes, as organisms with larger genomes will generate more reads. Amplicon sequencing using the 16S rRNA gene also suffers bias due to variability in 16S rRNA copy number [12]. Thus, marker based approaches using shotgun metagenomics sequencing data may provide the least biased relative abundance information for organisms in the community.

### Tools vary in several additional characteristics which may influence researcher’s choice

In addition to the class of method, there are many other characteristics which may affect the consideration of which method to use. For example, whether a method is available via a GUI, command line, or web server can be an important consideration, as is whether the method can also perform functional (gene function) classification, or how much memory and compute time the method requires. In addition, some methods are limited to certain groups of microbes. Some methods, such as AMPHORA2 [13], are limited to analysis of Bacteria and Archaea. Others, such as PhyloSift [14], can additionally predict Viruses and Eukaryotes. Furthermore, some methods continue to be supported while others are not, and some eventually become unavailable or difficult to access.

Another distinction that can be made is between methods which are rank-flexible, versus rank-specific. Rank-flexible methods vary the rank at which reads are predicted by classifying each read to the lowest taxonomic level at which the given method is confident. An example of a simple rank-flexible method is the lowest common ancestor (LCA) approach, first used by MEGAN [15]. This approach takes the set of taxa that the read hit in the similarity search (taking only those hits scoring within a threshold of the top hit), and assigns the read to the LCA of this set. In contrast, rank-specific methods give the same rank predictions for all reads.

### Clade exclusion is an important technique to evaluate how well methods will perform on environmental samples

Sequence similarity based methods perform very well when identifying query reads identical to genomes/sequences within the reference database that they search against. However, because the majority of microorganisms have not had their genome sequenced, in most environments many of the sequence reads that would be generated in a metagenomics experiment would be quite unrelated to any sequences that are in a reference database, or at minimum not identical [16]. Thus, one of the approaches used in the evaluation of taxonomic classifiers is clade-level exclusion. This involves removing all sequences from a database at a certain taxonomic level and then evaluating the ability to make predictions at higher taxonomic levels. For example, if performing species level exclusion for *Pseudomonas aeruginosa*, all *Pseudomonas aeruginosa* genome sequences would be removed from the reference database and/or models of the methods being evaluated. Then, the method’s ability to classify reads from *Pseudomonas aeruginosa* at higher taxonomic levels (i.e., *Pseudomonas*, *Pseudomonadaceae*, etc.) would be evaluated. Such clade exclusion methodology is one way to avoid obtaining artificially high accuracy levels caused by the problem of testing and training with identical data.

### The present work builds upon a previous evaluation performed without clade exclusion

There has been one previous evaluation of metagenomics bioinformatics methods reported that is not limited to examining a small set of tools with its own tool [17]. This study was an important first step in comparing many metagenomics classification tools; however, the microbial genomes used in the analysis were found in the reference databases and training sets of the methods evaluated. This means that the accuracy of the methods shown from the study will be considerably higher than when they are used to classify reads from organisms not in the reference databases or training sets. Samples from most environments, such as soil, ocean, and freshwater samples, are very diverse and the majority of organisms existing in these environments have not been characterized. The human gut is an environment in which intense research interest has resulted in substantial effort to sequence relevant microbes [18]; however, even in the human gut, it appears that the majority of species are not present in reference databases [19]. In addition, the previous comparison relied solely on *in silico* simulated reads. As sequence simulators cannot capture all of the factors that may affect read sampling in metagenomics, *in vitro* communities (i.e., samples of known bacterial cultures spiked into distilled water and sequenced) are an important complementary set of data to evaluate methods on. An unpublished study was recently made publicly available, which includes an evaluation using *in silico* evolved genomes [20]. This approach, with its artificially evolved sequences, complements the clade exclusion approach taken here where we use both computationally simulated and real sequences. One additional notable difference is that their evaluation looked only at the phylum level classifications, whereas this study looks at classifications at all taxonomic levels. Furthermore, they constructed their communities to contain only 5 % taxonomically novel (artificially evolved sequences). Therefore, the results are not comparable to our evaluations using clade exclusion where all of the sequences are from genomes not in the reference databases of the methods, and where performance is based on classification at all taxonomic levels rather than just at the phylum level.

In the present study, a variety of metagenomic taxonomic classification methods are evaluated on mock communities simulated both *in silico* and *in vitro* (distilled water spiked with known bacteria from pure culture, and sequenced). The performance of the methods in terms of their sensitivity, precision, and number of incorrectly predicted species are analyzed. In addition, the performance of the methods is compared as simulated read length is increased, and level of clade exclusion is varied. Methods evaluated more fully were chosen to encompass the range of types of methods available, as well as based on their popularity, and amenability to clade exclusion. We demonstrate how the accuracy of shotgun metagenomics classification methods varies widely. No one program clearly outperformed others in all evaluation scenarios, rather the results illustrate the strengths and weaknesses of different methods for different purposes—information critical for researchers to be aware of when performing their particular analysis.

## Methods

### Simulation of MetaSimHC and freshwater *in silico* and *in vitro* datasets

Two different microbial communities were used for this evaluation, both made up of diverse taxa for which completed genome sequences were available. The first was previously proposed as a “high complexity” dataset in [21], and will be referred to as MetaSimHC. This was chosen since it has been proposed to be a reference dataset for analysis of methods, and consists of diverse microbial species covering several phyla of both Bacteria and Archaea. The second was chosen with the aim of having a set of species commonly found in freshwater, suitable as a control for a watershed metagenomics project we participated in [22]. This was done by identifying species that were common among several publicly available freshwater datasets [23–25], and will be referred to as FW (freshwater). The organisms used in each of these datasets can be found in Table 1. Both of these datasets were simulated using MetaSim (version 0.9.5; [21]) at sequence lengths of 100, 250, 500, and 1000 bp, with each organism at 1X coverage. Although the sets of sequences of differing read length were generated independently, they are generated at 1X coverage so the effects of sampling only portions of genomes that are predicted particularly well or poorly should be mitigated. No error model was used, because there was not an error model for Illumina reads at the longer read lengths (500 and 1000), and we wanted to be consistent as read length was varied. Also, the *in vitro* dataset gives us data off of an actual sequencer which allows us to see how methods perform on data with real sequencing errors. Clade exclusion was performed at the level of species, genus, family, order, and class. The FW dataset was simulated both with MetaSim (FW *in silico*) and an *in vitro* mock community (FW *in vitro*). To construct the FW *in vitro*, the bacteria were grown up in pure culture, and then their DNA were extracted and spiked in equal concentrations into sterile, distilled water for sequencing. All complete bacterial and archaeal genomes were downloaded from NCBI on June 17, 2013, for the creation of databases and supervised models used in the different methods. The numbers of genomes left in the databases and training sets of the methods in the evaluation scenarios are shown in Additional file 1: Table S1. The datasets used in these evaluation scenarios have been deposited to the MG-RAST database and accession numbers can be found in Additional file 1: Table S2, and the number of reads simulated from each organism for the *in silico* datasets can be found in Additional file 1: Table S3. Note that while certainly test datasets could be constructed using a larger number of species, it is non-trivial to construct a similar *in vitro,* mock community dataset using a high number of species. We purposefully constructed our dataset to contain taxa with a variety of levels of divergence from one another, including closely related species (i.e. multiple species from the *Pseudomonas* genera). The latter helps evaluate the ability of methods to handle taxa prediction when closely related taxa are present.

**Table 1:** Microbes used in the 2 simulated mock communities

MetaSimHC^a^			Freshwater^b^ (FW) *in silico* and *in vitro*		
Genus	Species	Strain	Genus	Species	Strain
*Agrobacterium*	*tumefaciens*	C58	*Bacillus*	*amyloliquefaciens*	FZB42
*Anabaena*	*variabilis*	ATCC 29413	*Bacillus*	*cereus*	ATCC 14579
*Archaeoglobus*	*fulgidus*	DSM 4304	*Burkholderia*	*cenocepacia*	J2315
*Bdellovibrio*	*bacteriovorus*	HD100	*Escherichia*	*coli*	K-12
*Campylobacter*	*jejuni*	81–176	*Frankia*	*sp.*	CcI3
*Clostridium*	*acetobutylicum*	ATCC 824	*Micrococcus*	*luteus*	NCTC 2665
*Lactococcus*	*lactis*	SK11	*Pseudomonas*	*aeruginosa*	PAO1
*Nitrosomonas*	*europaea*	ATCC 19718	*Pseudomonas*	*aeruginosa*	UCBPP-PA14
*Pseudomonas*	*aeruginosa*	PA7	*Pseudomonas*	*fluorescens*	Pf-5
*Streptomyces*	*coelicolor*	A3(2)	*Pseudomonas*	*putida*	KT2440
*Sulfolobus*	*tokodaii*	str. 7	*Rhodobacter*	*capsulatus*	SB 1003
			*Streptomyces*	*coelicolor*	A3(2)

^a^MetaSimHC is a test dataset of 11 diverse microbial genomes covering several phyla of Bacteria and Archaea proposed in [21]

^b^Freshwater (FW) is a set of bacterial genomes found in previous freshwater metagenomics studies (see Methods)

Because there is such a large difference in microbial communities (e.g. soil versus acid mine drainage) in terms of number of organisms, which organisms are present, their taxonomic novelty, and diversity in terms of abundance distribution, it is not possible to simulate communities that will be appropriate for all environmental communities. This is why we suggest researchers test their own mock communities that approximate their expected community.

### Laboratory preparation and sequencing of the mock freshwater *in vitro* community

*Bacillus amyloliquefaciens* FZB42 (ATCC# 23842), *Bacillus cereus* (ATCC# 14579), *Escherichia coli* K12 (ATCC# 23716), *Micrococcus luteus* NCTC 2665 (ATCC# 4698), *Pseudomonas fluorescens* Pf-5 (ATCC# BAA-477), and *Pseudomonas putida* KT2440 (ATCC# 47054) were obtained as freeze-dried stocks and used per recommended protocol to start cultures in prescribed media. *Burkholderia cenocepacia* J2315 was cultured in Luria broth at 37 °C. *Frankia* sp. CcI3 was grown in liquid *Frankia* defined minimal medium (FDM) in stationary culture at 30 °C for 1 week. *Pseudomonas aeruginosa* UCBPP-PA14 was cultured in Luria-Bertani broth at 37 °C. *Rhodobacter capsulatus* SB 1003 was cultured on 0.3 % yeast extract, 0.3 % bactopeptone, CaCl_2_ (1 mM) and MgSO_4_ (1 mM) at 30 °C. *Streptomyces coelicolor* A3 was cultured in 0.5 % Tryptone, 0.3 % yeast extract, pH 7.1 at 28 °C for 1 week. For each of the strains of bacteria, after they were plated on the appropriate media, single colonies were picked. These were cultured overnight in 3 ml of appropriate media at the appropriate temperature (as above). *Frankia* sp. CcI3 and *Pseudomonas aeruginosa* UCBPP-PA14 were cultured for several days until they reached stationary phase. The other bacteria strains were fast growing, so the starter cultures were diluted 1:100, and grown with vigorous shaking (250 rpm) to saturation overnight. Genomic DNA was extracted from these cultures with the NucleoSpin Tissue kit from Macherey-Nagel according to manufacturer’s instructions. For Gram-positive bacteria, cells were pre-incubated with buffer containing 20 mg/ml lysozyme for an hour at 37 °C, followed by Proteinase K at 56 °C until complete lysis was obtained. The library was prepared using a Nextera XT DNA sample preparation kit following the manufacturer’s instructions. This library was sequenced with a MiSeq platform using a V2 500 cycles kit.

### Quality control of sequenced reads

Trimmomatic-0.25 [26] was used to (1) trim reads using a sliding window of 15 and PHRED quality score of Q < =20, followed by (2) checking if any of the last 5 bases had a Q < =5, and if so removing up to that base, and finally (3) filtering out any reads with length <85 bases. After quality control, there were 300,969 reads with an average length of 223 nucleotides.

### Evaluation of methods and metrics

Performance metrics used to evaluate different software are sensitivity, precision, taxonomic distance, and running time. Sensitivity and precision are calculated based on the numbers of true-positives (TP), false-positives (FP), and false-negatives (FN). True-positives are the number of reads assigned correctly, false-positives are the number of reads assigned incorrectly, and false-negatives are the number of reads unassigned. Sensitivity was calculated as TP/(TP + FN), and precision as TP/(TP + FP). Taxonomic distance was calculated from correctly assigned reads as the average number of ranks above the best possible rank the assignment could be made at, and running time as the number of minutes taken for the program to complete classification. For sensitivity, precision, and taxonomic distance, the values were averaged over all the species in the test dataset. This gave equal weighting to all of the species in the datasets; otherwise, the species with larger genomes (which have more reads) would have a larger influence on the scores. For the *in silico* datasets, reads were categorized as correctly assigned (TP) if they classified to a node (taxonomic rank) that was anywhere in the path from the correct species to the superkingdom level (e.g. Bacteria) of the NCBI taxonomic tree, and as incorrect if the read was assigned to a node that was not in this path. In the case where overpredictions were considered correct, the taxonomic level that was used to determine if a read was classified correctly was the best possible correct level that could be predicted. For example, under species clade exclusion, reads would still be classified as correct if they were in the correct genus but classified to an incorrect species. Although most of the methods evaluated were rank-flexible in their predictions, RITA and PhymmBL are rank-specific, and thus were only shown for the evaluation where overpredictions were considered correct. Although RITA does have a rank-flexible mode, it requires having 16S rDNA profiles of a community. PhymmBL provides a confidence score which in theory could provide a cut-off for which rank to assign the reads; however, we would have had to choose the cut-offs ourselves, and previous researchers have found confidence scores to be high for a false positive dataset [27]. MG-RAST was evaluated due to the popularity of the method, but because it does not allow the user to create custom clade exclusion reference databases, it is an example of a method where we were only able to evaluate it without clade exclusion.

Additional file 1: Table S4 lists the version numbers of all of the methods evaluated. All methods were run with default parameters except for filtered Kraken [28] which was run using the kraken-filter script with a threshold of 0.20, which moves assignments up to successfully higher levels of the taxonomic tree until the threshold is reached. This separate analysis was done because we noticed that Kraken was tending to overclassify reads and there was an option that would help assign reads with greater confidence. Note that some methods have variations in the way they can be run. For example, some methods can take a variety of similarity search programs as input, or have the option to utilize paired-end sequence read information. In some cases these variations had relatively small differences in sensitivity, precision, and taxonomic distance of methods, and in these cases only one of the variants was presented in the figures to be concise. Briefly, MEGAN4 [29] has the option to allow the use of paired-end information from sequence reads, and the paired-end version is presented; MetaPhyler [30] can use BLASTX, BLASTN, or a combination of the results, and the results for the BLASTX/BLASTN combination are presented; MEGAN4 and DiScRIBinATE [31] have the option of taking results as input from either RAPSearch2 [32] or BLASTX, and the RAPSearch2 versions are presented. RAPSearch2 is an alternative to BLAST, which we found to run over 30 times faster than BLASTX, with comparable accuracy (see Results).

## Results

Table 2 provides an overview of methods and their features, grouped by their class. Note that it does not include all methods available, and there are more methods being continually published. Included is the number of citations each method has received, to give an indication of how much of an influence or use each method has. However, it should be noted that several of the methods have capabilities beyond just classification, such as comparisons between samples and visualization, and thus may be cited when used for purposes other than classification. Also, it is worth noting that methods that were published earlier may be highly cited, yet newer methods often improve upon their strategies. As discussed below, even with accuracy assessment aside, the different method properties can have different advantages under certain analysis scenarios and so are summarized here. Notably, many methods cannot undergo full, robust evaluation with clade exclusion, since their reference databases cannot be manipulated, and so methods chosen for full evaluation of the accuracy were limited to ones that allowed it.

**Table 2:** List of metagenomics sequence classification methods and their characteristics sorted by class of method

Method name	Class of method	Sequence alignment method/Composition method	Standalone^a^/Web server	Most recent year published (first time published)^b^	Functional classification if applicable	References	Number of citations^c^
MEGAN4	Similarity	MEGABLAST, BLASTN, BLASTX, RAPSEARCH2 [32] / N/A	Yes/No	2011 (2007)	KEGG, SEED	[15, 29, 45–47]	1089
MG-RAST	Similarity	BLASTN, BLAT / N/A	No/Yes	2008	SEED, NOG, COG, KEGG	[48]	691
CAMERA	Similarity	All 6 BLAST programs / N/A	No/Yes	2007 (2011)	Pfam, TIGRFAM, COG, KOG, PRK	[49, 50]	324
CARMA3	Similarity	BLASTX, HMMER3 [51] / N/A	Yes/Yes	2011 (2008)	GO	[41, 52, 53]	201
WebMGA	Similarity	FR-HIT [54] / N/A	No/Yes	2013	Pfam, TIGRFAM, COG, KOG, PRK, GO	[55]	54
DiScRIBinATE (SOrt-ITEMS)^d^	Similarity	BLASTX, RAPSEARCH2 / N/A	Yes/No	2010 (2009)	N/A	[31, 56]	48
Ray Meta	Similarity	Exact match k-mers / N/A	Yes/No	2012	N/A	[57]	34
Kraken	Similarity	Exact match k-mers / N/A	Yes/No	2014	N/A	[28]	15
RTM	Similarity	k-mers / N/A	Yes/Yes	2012	KEGG	[58]	12
Genometa	Similarity	Bowtie [59], BWA [60] / N/A	Yes/No	2012	N/A	[61]	7
LMAT	Similarity	Exact match k-mers / N/A	Yes/No	2013	N/A	[62]	6
Sequedex	Similarity	Exact match k-mers / N/A	Yes/No	2012	N/A	[63]	5
MetaBin	Similarity	BLASTX, BLAT / N/A	Yes/Yes	2012	COG	[64]	4
TAMER	Similarity	MEGABLAST / N/A	Yes/No	2012	N/A	[65]	4
metaBEETL	Similarity	Direct comparison of compressed text indices / N/A	Yes/No	2013	N/A	[7]	2
SPANNER	Similarity	BLASTP / N/A	Yes/No	2013	N/A	[66]	2
GOTTCHA	Similarity	BWA / N/A	Yes/No	2015	N/A	[67]	0
CLARK	Similarity	k-mers / N/A	Yes/No	2015	N/A	[68]	0
MLTreeMap	Marker	BLASTX / N/A	Yes/Yes	2010 (2007)	4 Enzyme families	[69, 70]	206
AMPHORA2	Marker	HMMER3 / N/A	Yes/Yes	2012 (2008)	N/A	[13, 71, 72]	190
MetaPhlAn	Marker	MEGABLAST, Bowtie2 [73] / N/A	Yes/Yes	2012	N/A	[11]	114
MetaPhyler	Marker	BLASTN, BLASTX / N/A	Yes/No	2011	N/A	[30]	42
mOTU	Marker	HMMER3 / N/A	Yes/Yes	2013	N/A	[19]	24
Phylosift	Marker	LAST, HMMER3 / N/A	Yes/No	2014	N/A	[14]	18
phymmBL	Hybrid	MEGABLAST / IMM	Yes/No	2011 (2009)	N/A	[6, 74]	182
RITA	Hybrid	Pipeline of BLAST variations / NB	Yes/Yes	2012 (2011)	N/A	[75, 76]	38
SPHINX	Hybrid	BLASTX / k-means	No/Yes	2010	N/A	[10]	17
TaxyPro	Hybrid	CoMet web server / Mixture model	Yes/No	2013	Pfam	[77]	3
TWARIT	Hybrid	BWA short read alignment [60] / k-means	No/Yes	2012	N/A	[78]	2
PhyloPythiaS	Composition	N/A / SVM	Yes/Yes	2011 (2007)	N/A	[30, 79, 80]	269
TACOA	Composition	N/A / k-NN	Yes/No	2009	N/A	[33]	65
NBC	Composition	N/A / NB	Yes/Yes	2011 (2008)	N/A	[81, 82]	35
RAIphy	Composition	N/A / RAI	Yes/No	2011	N/A	[83]	18
ClaMS	Composition	N/A / DBC signature	Yes/No	2011	N/A	[84]	10
INDUS	Composition	N/A / k-means	No/Yes	2011	N/A	[85]	8
TAC-ELM	Composition	N/A / Neural Network	Yes/No	2012	N/A	[86]	5
MetaCV	Composition	N/A / CV	Yes/No	2013	KEGG	[87]	4
GSTaxClassifier	Composition	N/A / Bayesian	No/No	2010	N/A	[88]	2

*N/A* not applicable, *IMM* interpolated Markov model, *NB* naive Bayes, *SVM* support vector machine, *k-NN* k-Nearest Neighbour, *RAI* relative abundance index, *DBC signature* de Bruijn chain signature, *CV* composition vector

^a^Standalone refers to whether the program can be run locally

^b^Some methods have had several publications, with later publications regarding improvements on functionality. In these cases the most recent publication was listed, with the first time the method was published in brackets

^c^Number of citations is based on Web of Science as of April 21, 2015

^d^DiScRIBinATE is the successor for SOrt-ITEMS so they were included in the same row

### Several methods vastly overestimate the number of species present

To assess accuracy, first the quality of the assignments made by different methods was examined with no clade exclusion, so that as many representative methods could be comparatively examined as possible. The sensitivity, precision, and taxonomic distance (Additional file 2: Figures S1 and S2) were computed on the MetaSimHC dataset with no clade exclusion. Results were as expected, with all methods generally showing a relatively high sensitivity and precision. The exceptions are TACOA [33], which is known to perform poorly on short reads, and MetaPhyler, which is a marker based method and thus only classifies a small proportion of the reads, resulting in low sensitivity (but high precision). Next, the numbers of incorrectly predicted species, based on different thresholds of percentage abundance in the predicted community were tabulated (Additional file 1: Table S5). It is notable that several methods greatly overpredict the numbers of species present, considering that the sequences the methods are trying to classify exist in the reference databases or training sets. Under genus clade exclusion conditions (Additional file 1: Table S6), the number of incorrectly predicted species increases further for any method that makes incorrect predictions at the examined taxonomic level.

### Sensitivity and precision vary widely between methods, with sensitivity generally decreasing at higher levels of clade exclusion and increasing with read length

**Fig. 1:**
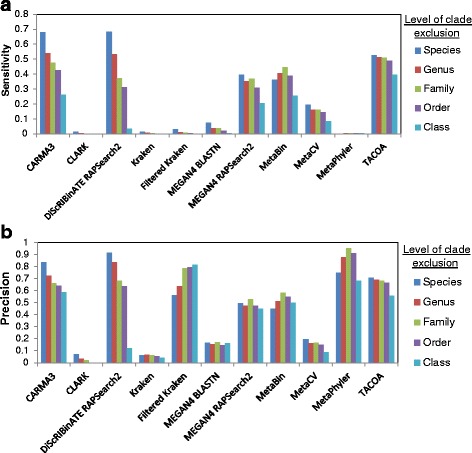
Performance as clade exclusion level is varied. Sensitivity (**a**) and precision (**b**) on the MetaSimHC dataset of simulated 250 bp reads. There is a wide range of variability in the sensitivity and precision of the methods with sensitivity tending to decrease as the level of clade exclusion moves from species to class. Performance is calculated based on proportion of reads appropriately assigned and averaged per genome (see Methods)

**Fig. 2:**
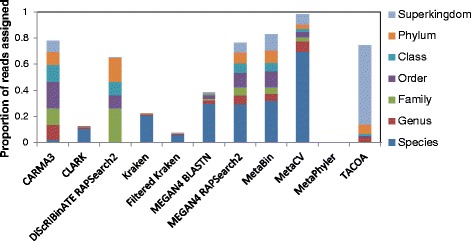
Distributions of assignments to taxonomic ranks. Proportion of reads assigned at each taxonomic rank on the MetaSimHC dataset of simulated 250 bp reads under genus clade exclusion (includes both correct and incorrect assignments). Although the lowest possible correct rank is family, many methods still classify the majority of reads at the species level. CARMA3 and DiScRIBinATE are slightly more conservative, classifying a large number of reads at the family or order levels, whereas TACOA is extremely conservative, classifying the majority of the reads at the superkingdom level

The quality of the assignments made by the different methods was further examined under clade exclusion scenarios at different taxonomic levels. Sensitivity and precision were computed on the MetaSimHC dataset (Fig. 1) and found to vary notably. To examine in greater detail what led to the differences in sensitivity and precision of these methods, the taxonomic distance for each method was evaluated (Additional file 2: Figure S3). Furthermore, the proportion of reads assigned at each taxonomic rank was determined. An example of the results under the genus clade exclusion scenario is shown in Fig. 2, with the data for the rest in Additional file 3. Additionally, the numbers of reads miss-assigned and correctly assigned or overpredicted for each rank were compiled (genus clade exclusion Additional file 2: Figure S4, the rest of the data in Additional file 4). Many of the methods assign a considerable proportion of reads to the species level, when species level assignment is impossible since they are excluded from the database. Also notable is that TACOA assigns the majority of reads to the superkingdom level, so the method will be of limited use for those interested in more specific taxonomic ranks, at least at these shorter read lengths.

**Fig. 3:**
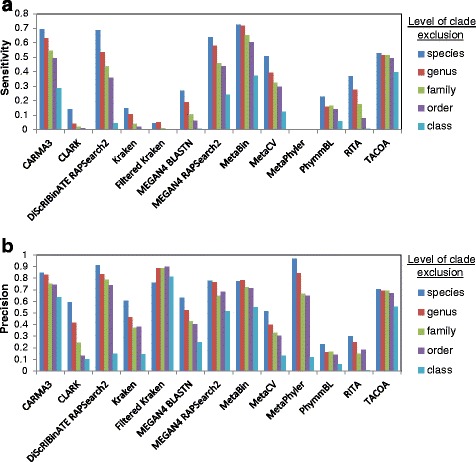
Performance as clade exclusion level is varied with overpredictions (see Methods for details) classified as correct. Sensitivity (**a**) and precision (**b**) on the MetaSimHC dataset of simulated 250 bp reads. Methods such as MEGAN4 which classify many reads at lower taxonomic levels see a considerable increase in performance, whereas more conservative methods such as CARMA3 see only a slight improvement. Performance is calculated based on proportion of reads appropriately assigned and averaged per genome (see Methods)

In some cases, overpredictions (e.g. predictions made to an incorrect species in the correct genus) are less problematic than incorrect predictions (e.g. predictions made to an incorrect genus). Thus, sensitivity and precision were recalculated after reclassifying overpredictions as correct classifications (Fig. 3). There was notable increase in sensitivity and precision for methods such as MEGAN4 and MetaBin which are less conservative in their predictions. For more conservative methods such as CARMA3 and DiScRIBinATE, there was little change.

The changes in sensitivity, precision, and taxonomic distance as read length increased was then examined. This was done on the MetaSimHC dataset (Additional file 2: Figure S5). Sensitivity followed the expected trend of increasing along with read lengths; however, precision and taxonomic distance showed no clear trend and remained relatively unchanged.

### Analysis of the FW dataset reveals similar performance between *in vitro* data and *in silico* data, and between the FW and MetaSimHC datasets

**Fig. 4:**
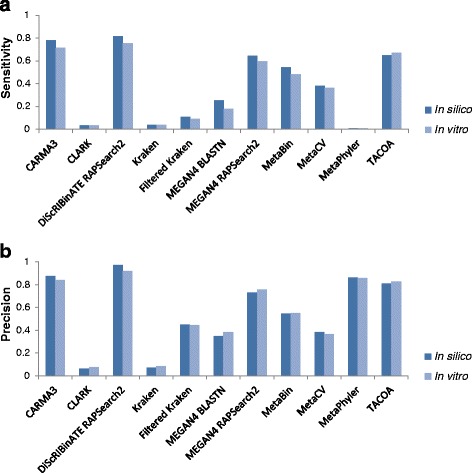
Performance of FW *in silico* versus FW *in vitro*. Sensitivity (**a**) and precision (**b**) of methods on the FW dataset comparing the performance on the *in silico* community versus the *in vitro* community under species clade exclusion. The results are similar between the *in vitro* and *in silico* communities, demonstrating that methods appear to be relatively robust to real Illumina sequencing errors for this simple community. Performance is calculated based on proportion of reads appropriately assigned and averaged per genome (see Methods)

**Fig. 5:**
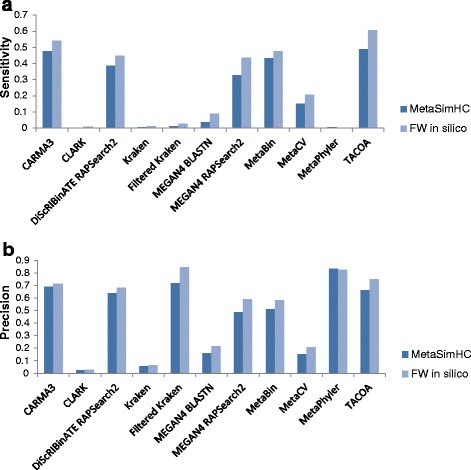
Performance of MetaSimHC compared to FW *in silico*. Sensitivity (**a**) and precision (**b**) of methods on the MetaSimHC dataset compared to the FW *in silico* of simulated 250 bp reads. Values are averaged over all levels of clade exclusion from species to class. Although the microbes in the dataset changed, the relative performance of the methods remains very similar. Performance is calculated based on proportion of reads appropriately assigned and averaged per genome (see Methods)

A comparison between the FW *in silico* versus *in vitro* datasets is illustrated in Fig. 4 under species clade exclusion, and in Additional file 2: Figure S6 without clade exclusion. For the *in vitro* dataset, as it is not possible to determine which read absolutely should be associated with which organism in the mock microbial community, a hit to any of the taxa in the FW dataset was considered correct. In addition, this meant the sensitivity, precision, and taxonomic distance was based on all of the reads classified rather than averaged over all taxa. The results are similar between the *in vitro* and *in silico* communities, suggesting that for this simple community the methods evaluated are relatively robust to Illumina sequencing errors with the sequencing technology used. A comparison of results between MetaSimHC and FW *in silico* revealed that the relative performance of methods remained similar when analyzing these two different datasets (Fig. 5). Additionally, the numbers of incorrectly predicted species, based on different thresholds of percentage abundance in the predicted community, were again tabulated for the *in vitro* data (Table 3). Many of the methods incorrectly predict hundreds of species, with MetaCV incorrectly predicting 1226 species, although after filtering out low abundance predictions the numbers of incorrect predictions were drastically reduced. Under genus clade exclusion conditions (Additional file 1: Table S7), the number of incorrectly predicted species increases further, and even after filtering out low abundance predictions there were sometimes considerable numbers of false species predictions. The number of incorrectly predicted species is higher for the *in vitro* data relative to the *in silico* data (Table 4). The greater number of incorrectly predicted species is particularly notable in some methods that perform very well on the *in silico* data such as MEGAN4 BlastN, which goes from 0 incorrectly predicted species to 110. The performance for each of the component genomes on all *in silico* datasets is provided in Additional file 5.

**Table 3:** Number of correctly and incorrectly predicted species^a^ for different thresholds^b^ without clade exclusion. Some methods vastly overpredict the number of species, even when the true number of species is low (in this case the true number of species is 11)

	No cutoff^b^		Cutoff > 0.01 %^b^		Cutoff > 0.1 %^b^		Cutoff > 1 %^b^	
Method	Correct	Incorrect	Correct	Incorrect	Correct	Incorrect	Correct	Incorrect
CARMA3	11	56	11	4	11	0	10	0
CLARK	11	364	11	25	11	5	11	0
DiScRIBinATE RAPSearch2^c^	N/A	N/A	N/A	N/A	N/A	N/A	N/A	N/A
Kraken	11	327	11	25	11	5	11	0
Filtered Kraken	11	14	11	1	11	0	11	0
MEGAN4 BlastN	11	110	11	19	11	3	9	1
MEGAN4 RAPSearch2	11	183	11	41	11	1	9	1
MetaBin	11	561	10	77	10	6	10	1
MetaCV	11	1226	11	232	11	6	10	1
MetaPhyler	11	9	11	9	11	5	7	1
PhymmBL^c^	N/A	N/A	N/A	N/A	N/A	N/A	N/A	N/A
RITA	11	466	10	80	10	10	10	1
TACOA^c^	N/A	N/A	N/A	N/A	N/A	N/A	N/A	N/A
MG-RAST best hit	11	927	10	180	10	36	10	8
MG-RAST LCA	11	476	11	69	11	5	11	1

^a^Using the FW *in vitro* dataset of sequenced reads from 11 species

^b^A cutoff of > × %, for example 0.01 %, would indicate that only species with a predicted abundance of at least x % of the total set of predictions were considered. Correctly predicted species are any of the 11 species that were used to simulate the reads in the dataset, whereas any other predicted species was incorrect

^c^These methods do not predict to the species level at this read length (they require longer read lengths). See additional analyses at other levels of clade exclusion

**Table 4:** Number of incorrectly predicted species^a^ for different abundance thresholds^b^ without clade exclusion. Fewer incorrectly predicted species are predicted with the *in silico* data that does not contain errors versus the *in vitro* data containing sequencing errors (Table 3)

	No cutoff^b^		Cutoff > 0.01 %^b^		Cutoff > 0.1 %^b^		Cutoff > 1 %^b^	
Method	Correct	Incorrect	Correct	Incorrect	Correct	Incorrect	Correct	Incorrect
CARMA3	11	41	11	3	11	1	11	1
CLARK	11	0	11	0	11	0	11	0
DiScRIBinATE RAPSearch2^c^	N/A	N/A	N/A	N/A	N/A	N/A	N/A	N/A
Kraken	11	0	11	0	11	0	11	0
Filtered Kraken	11	0	11	0	11	0	11	0
MEGAN4 BLASTN	11	0	11	0	11	0	10	0
MEGAN4 RAPSearch2	11	92	11	29	11	1	10	0
MetaBin	11	286	11	41	11	3	11	0
MetaCV	11	0	11	0	11	0	11	0
MetaPhyler	10	12	10	12	10	8	7	3
PhymmBL^c^	N/A	N/A	N/A	N/A	N/A	N/A	N/A	N/A
RITA	11	0	11	0	11	0	11	0
TACOA^c^	N/A	N/A	N/A	N/A	N/A	N/A	N/A	N/A
MG-RAST best hit	10	646	10	136	10	26	10	6
MG-RAST LCA	10	300	10	54	10	8	9	3

^a^Using the FW *in silico* dataset of sequenced reads from 11 species

^b^A cutoff of > × %, for example 0.01 %, would indicate that only species with a predicted abundance of at least × % of the total set of predictions were considered

^c^These methods do not predict to the species level at this read length (they require longer read lengths). See additional analyses at other levels of clade exclusion

### There is substantial variation in the computational cost of different methods

To evaluate how long the various methods took to run, 22,000 reads of 100, 250, 500 and 1000 bp, and an additional 44,000 reads of 250 bp were simulated using the MetaSimHC dataset. The time taken by the methods to complete an analysis of these sequences varied widely, and nearly all methods scaled roughly linearly with both read length and number of reads on our datasets (Additional file 2: Figure S7). Sequence similarity based methods that rely on BLASTX take considerably longer than all other methods except TACOA, taking over 24 h for just 22,000 reads of 250 bp under the CPU conditions in the test (one Intel Xeon E5-2660 2.2 GHz CPU and 282 GB of RAM). At the other extreme, Kraken and CLARK took less than 1 min to classify all of the reads.

## Discussion

All of the methods analyzed performed very well in terms of sensitivity and precision when the query sequences were in the reference databases (i.e. when there was no clade exclusion). Of course, this type of analysis would be expected to give potentially artificially high accuracy values since one is essentially evaluating using test data identical to the reference/training data. Under this type of analysis scenario, the more informative metrics to examine are taxonomic distance and the number of incorrectly predicted species. Notably, several methods substantially overpredicted the number of species present in the simulated communities. This included popular methods such as MG-RAST and MEGAN4. However, most of these incorrectly predicted species are predicted at a very low abundance. By setting a threshold to filter out low abundance predictions, the number of incorrect predictions can be considerably reduced. The thresholds presented here are not intended as suggestions, but rather to demonstrate the principle of using thresholds to filter out incorrect predictions. Microbial communities in certain environments are very complex, such as those found in soil [34]. These environments, which are very diverse and contain a large number of organisms, would have a large proportion of the microbes found at less than 1 % of the total abundance of the community, and thus a 1 % filtering threshold would filter out many of the microbes actually in the metagenome. If thresholds are used, they should ideally be chosen based on a mock community control that reflects the anticipated level of diversity and complexity expected in the metagenomics analysis being performed. If the goal is to choose thresholds based on relative abundance, genome size of the organisms would also be useful to take into account. Otherwise, if two organisms are present in the community at low levels but one organism’s genome is much bigger, the organism with the smaller genome may get filtered out while the organism with the larger genome does not, due to greater number of reads from the larger genome. It is important for researchers doing metagenomics projects to know the level of precision of the method that they are using to have an idea of how well they can trust the taxa predicted at lower abundance. There is a trade-off between finding all of the taxa that exist in the sample, and confidence in the prediction of the taxa. Two ways to adjust this trade-off are to choose a more precise (conservative) method, or to alter the minimum abundance threshold, with only the taxa over this abundance threshold being reported. Some methods already have a way of choosing this threshold. For example, MEGAN4 by default requires at least 5 reads to hit a taxon before the taxon is reported. The reads that are initially assigned to a taxon with less than the chosen threshold number of reads are then pushed up the taxonomy until they reach a taxon with a number of reads assigned to it that is over the threshold. However, when many reads are analyzed, overprediction will still occur and we have found for our analyses that it is necessary to use an additional threshold for removal of low abundance reads that are likely false predictions for such methods. Ideally this threshold may be chosen in part from an analysis of an *in vitro* mock community sample—an important experimental control in any metagenomics analysis. Such evaluation of methods using real sequence data also acts as an additional important control regarding other aspects of metagenomics sequencing pipelines.

As demonstrated in Fig. 1, the sensitivity and precision of methods vary dramatically. Methods show a general trend of decreasing sensitivity as the rank of clade exclusion increases. This is expected as the sequences left in the database will become increasingly divergent, and the scores of the matches, if any, will decrease. There is a notable decrease in performance for methods relying on sequence composition or nucleotide-based BLASTN similarity searches, versus the protein/amino acid sequence-based BLASTX and RAPSearch2 similarity based methods. This confirms what has been reported previously, that sequence composition based methods have lower performance than sequence similarity based methods at shorter read lengths [6]. BLASTN is likely outperformed by amino acid-based similarity approaches under clade exclusion because nucleotide sequence search is well known to be less sensitive for more divergent sequences due to its lower number of different characters (4 bases versus the 20 amino acids).

The differences in performance between methods can be partially explained by the distribution of taxonomic ranks that they assign reads to. As seen in Fig. 2, CARMA3 and DiScRIBinATE are assigning reads more conservatively; that is, they are assigning much fewer reads to the lower taxonomic ranks. Many of these lower level predictions of other methods are in fact overpredictions, as demonstrated by their large increases in sensitivity and precision between Figs. 1 and 3. Due to the way we evaluated methods, the most conservative methods will show the highest sensitivity and precision, but may not be making classifications at specific enough taxonomic ranks to be useful. TACOA, for example, shows high sensitivity and precision, yet makes classifications at very high taxonomic ranks that would not be useful for most researchers.

Not surprisingly, the sensitivity increases for methods as read length increases. The most dramatic increase appears to be between read lengths of 100 and 250 bp. Thus, when choosing a sequencing technology, it may be important to try and obtain a sequence read length of at least around 250 bp. The precision and the taxonomic distance of methods remained relatively unchanged. This was likely due to any increased performance in precision and taxonomic distance offset by additionally classified reads (as seen by the increase in sensitivity) with greater dissimilarity to sequences in the databases of methods, which would have poorer performance in terms of precision and taxonomic distance.

Our comparison of the *in silico* to the *in vitro* freshwater community showed similar results in terms of relative performance of the methods. This gives us some confidence in our results of the other *in silico* simulations, as well as demonstrating the robustness of the evaluated methods to real sequence errors for this simple community. However, this would not necessarily generalize to more diverse communities, or other sequencing technologies. The sensitivity and precision of the methods followed the trends seen in the MetaSimHC *in silico* evaluation, although filtered Kraken showed somewhat lower relative precision. Upon further analysis, this appeared to be due to the nature of the way precision was calculated in this comparison. For the comparison to be done fairly between the *in silico* and *in vitro* community, the metrics were based on all reads rather than the average for all organisms. Filtered Kraken seemed to stand out in that for most organisms it classified few of the reads, and the ones it classified were mostly correct. However, for two organisms (*E. coli* and *B. cereus*), the majority of the reads were classified incorrectly. This means that because more of the reads of *E. coli* and *B. cereus* were classified than the other organisms, their (mostly inaccurate) classifications had a relatively large influence on the precision. The numbers of genomes/taxa in the mock communities was small, relative to the anticipated number of species in most real metagenomic analyses, so abnormal results from individual genomes could have a large impact on the results, as seen here with filtered Kraken. It is also notable that *E. coli* and *B. cereus*, mainly due to historical reasons, come from regions of the taxonomic tree that are not reflective of the typical case for many environments; genomes with high sequence similarity and composition in this part of the tree are classified as the same species, whereas if they were found in other parts of the tree they would be classified as different species or genera [35, 36]. Thus, species that are not yet discovered will not be classified in a similar manner to the genomes in *Escherichia* or *Bacillus*, and so the performance of methods on these genomes likely does not reflect performance on as yet undiscovered microbes in metagenomics samples. However, it must be emphasized that there is no one mock community dataset that can best evaluate all metagenomics software. Key is for researchers to design mock communities for evaluation that are suitable for their experiment, and use this published analysis to appreciate the types of issues they should watch out for.

The differences we saw in computational cost of the methods were substantial. Although we only ran a few small test datasets of thousands of reads, we were able to clearly show very large differences in computational cost of the methods. Current metagenomics datasets often include millions of reads; without access to large amounts of compute power, many researchers will not find it practical to utilize BLASTX based methods for Illumina sequence sized data sets as are currently produced. The need for a more rapid alternative is already being addressed by such methods as RAPSearch2 [32], LAST [37], PAUDA [38], and DIAMOND [39]. Notably, RAPSearch2 shows similar, or in some cases even increased, performance relative to the same methods using BLASTX, while requiring much less time to run (over 30x faster in our analyses). Many methods provide the option of running multiple threads, so access to additional processors will allow the methods to run substantially quicker. Furthermore, for most methods reads are classified independently from one another, so files of reads can be broken up into multiple smaller files and each file run on a separate processor, and the results of the classifications combined. In addition to computational cost, the amount of RAM used by different methods varies considerably. Both Kraken and CLARK require large amounts of RAM, but do provide reduced standard databases for users with low-memory computing environments (known as MiniKraken and Clark-*l*). Certain methods also allow users to adjust settings to allow trade-offs between speed, accuracy and RAM usage, such as the sampling factor value in CLARK. A final consideration of computational resources when choosing a method is the amount of disk space that a method requires. The databases used by some methods require relatively large amounts of disk space, such as the standard database of Kraken which requires at least 160 GB of disk space. Another aspect that may affect method choice is the relative ease of generating new databases for the methods. Certain methods rely on the results of a similarity search, and expanding the database is a relatively simple process of generating a new database for that similarity search, such as BLAST. However, other methods may require substantial computational resources that researchers may not have access to. For example, the authors of GOTTCHA state that the creation of a database from the 2500 prokaryotic genome projects available in 2012 required 2 TB of RAM. Other methods, such as many online only methods, do not even allow the modification/expansion of the database.

Protein sequence similarity-based methods (e.g. BLASTX, RAPSearch2) perform very well in clade exclusion scenarios but do not perform as well as nucleotide based methods when there is no clade exclusion. This is likely because a proportion of microbial genome sequence (commonly around 6–14 % [40]) are non-coding. Protein similarity-based methods still have a relatively high sensitivity, generally >0.94 and, as noted in [41], this is due to many reads overlapping at least partially with a coding region. This explanation makes sense with our finding that as read length is increased, sensitivity of the aforementioned methods increases (from 0.94 at read lengths of 100 to 0.99 at read lengths of 1000 nucleotides for MEGAN4 BLASTX on the MetaSimHC dataset), as it would be less likely that a longer read would cover only non-coding regions. A quick examination of these incorrectly classified reads confirmed that they were the non-coding regions of the genomes, in many cases rRNA genes.

The results presented should guide researchers to the choice of method that best fits their research question and computational resources. Clearly, certain methods perform well in certain situations. Kraken, Filtered Kraken, and MEGAN4 BLASTN perform exceedingly well when there is no clade exclusion, yet their sensitivity is low when there is clade exclusion. However, filtered Kraken classifies only a small percentage of reads when the species present in the dataset is not in the database. For example, filtered Kraken classifies less than 8 % of the reads under genera exclusion (Fig. 2). A strategy researchers may therefore use is to take their dataset and first run it on filtered Kraken, followed by running the reads not classified by filtered Kraken on a more conservative method such as DiScRIBinATE RAPSearch2. Filtered Kraken would classify the reads from genomes in the reference database, while leaving the majority of reads from genomes not in the reference database unclassified. Then, DiScRIBinATE RAPSearch2, which will assign a much greater proportion of reads from genomes not in reference databases, could be run on the unclassified reads. If a conservative method such as DiScRIBinATE RAPSearch2 is run alone, it may miss many of the assignments of known genomes to the species rank, due to its tendency to make assignments at higher ranks. However, in some cases, such as when analyzing less well characterized microbiomes (such as in water versus human feces) the use of such conservative methods could be entirely appropriate. The pipeline idea of combining methods is integrated into some methods like RITA, which first identifies a highest-confidence set of predictions, then subjects the sequences not yet classified to a series of downstream classification steps. CARMA3 performs well in both the no-clade exclusion scenario (with a small taxonomic distance, classifying many reads to the species level) as well as the clade exclusion scenario. However, CARMA3 takes a considerable time to run, and may not be computationally feasible for those with large datasets and without access to notable compute power. Another technique involving combining methods would be to use multiple methods and look for consistent assignments among methods [27]. Depending on the type of analysis, this could increase precision and confidence in the assignments, although at the cost of sensitivity in most cases and run time (due to running multiple methods).

The test datasets used in this evaluation are limited in their complexity and diversity, as well as the number of reads simulated. For example, millions of reads are often sequenced for metagenomics samples, while our datasets were smaller, containing tens to hundreds of thousands of reads. Furthermore, many environments sampled are far more complex and diverse, containing a much larger number of microbes with varying relative abundance, such as soil or the human gut. Our analyses were also either on *in silico* simulated communities or communities sequenced with a single sequencing technology. The aim of this research was not to recommend any specific method, but to raise awareness of the advantages and disadvantages of different methods and issues in metagenome analyses. This evaluation highlights that there are large differences in methods on even the relatively simple communities used for our datasets, such as number of organisms predicted, sensitivity and precision, how specific the classifications tend to be (taxonomic rank), and computational resources required to run. However, other factors such as the diversity and microbes present in a community, and the sequencing technology used, will also affect the performance of the methods. Additionally, certain tools may have advantages and be particularly useful for specific environments. For example, some tools contain genomes in their databases that are not present in RefSeq, while most methods use RefSeq exclusively for their databases. An example of this is MetaPhlAn, which includes many draft genomes from the larger Human Microbiome Project (HMP) [42], and thus may be particularly useful for human microbiome samples. Metagenomics as a field is expanding rapidly. New tools are needed to classify the sequences obtained from these studies. There is a large need, and lots of interest in this, as evidenced by the large number of methods released over the past few years. However, it is non-trivial to perform an evaluation of methods. This is due to the sheer number of metagenomic methods available, the difficulty in setting up some of these methods, and the challenge in performing robust evaluation techniques such as clade exclusion or leave-one-out evaluation. Furthermore, methods only available on the web are generally unable to be thoroughly evaluated as in many cases they do not allow the use of custom reference databases or training sets, and sometimes limit the number of reads that can be uploaded. To address these difficulties, an initiative called the Critical Assessment of Metagenomic Interpretation (CAMI) has been initiated [43]. This community-led initiative will have researchers run their own methods on data sets made up of unpublished microbial genomes. This will be a valuable contribution to methodology assessment, but researchers are still encouraged to use mock microbial communities as controls for their own particular analyses, especially mock communities that reflect the types of microbes, diversity, and complexity they expect to see in their study. While CAMI will provide a useful additional comparative evaluation of methods, one should always perform a metagenomics analysis using appropriate controls to best refine methodology and any threshold cutoffs suitable for the specific analysis needs.

Another issue is that there does not seem to be a consensus on the way to evaluate performance. Some researchers consider classification of a read to a taxonomic level more specific than what is correct (e.g. a novel *Escherichia* species being assigned to *Escherichia coli* rather than *Escherichia*) as assigned correctly (e.g. [28]). Other researchers, however, classify these overprediction assignments as false positives or mispredictions (e.g. [31]). Depending on the research goal, one may prefer a more liberal or conservative method. For example, if a researcher is interested in comparing the genera in one metagenomics sample to another sample, overpredictions that are incorrect at the species level will not matter if they are correct at the genera level. The more conservative method may assign the same reads to the family level, and will thus completely miss the relevant taxa. On the other hand, if a researcher is interested in taking all of the predictions at all taxonomic ranks, they may make erroneous conclusions that a specific species is increased in one sample over another if it is just an overprediction. It should also be stressed that many methods allow flexibility in the parameters used, so it may be possible to tune a method to be more or less conservative. However, some parameters cannot be changed, and there are fundamental differences in the ways reads are classified by different methods. For example, MEGAN4 and MG-RAST make assignments based on bit-score as the sole parameter for judging significance. Other methods, such as DiScRIBinATE, CARMA3, and MetaPhyler, employ additional measures such as alignment parameter thresholds and/or a reciprocal BLAST search step, which have been shown to improve the accuracy of taxonomic assignments in certain scenarios [44]. For example, using these methods a read from a novel *Pseudomonas* species with a single hit over the bit-score threshold to *Pseudomonas aeruginosa* may not align well enough to be assigned to the species level based on the additional alignment parameters, and thus could be assigned correctly to *Pseudomonas*. However, in MEGAN4 or MG-RAST the read would pass the bit-score threshold and because there were no other hits, it would be assigned directly to *Pseudomonas aeruginosa*.

Again, careful examination of controls (like an *in vitro* mock community sequenced alongside metagenomics samples) may provide insight into the best method to use and suitable threshold cutoffs for low abundance reads, especially if that mock community includes a suitable level of diversity and/or includes species expected in the metagenomics analysis. Developers of new methods are encouraged to enable their method to be evaluated using customized reference datasets, including clade exclusion-based analysis, to enable robust analysis of their method.

## Conclusions

There has been a real need for a comprehensive evaluation of metagenomics classification methods, due to the notable number of new methods being released. In this case we have focused on taxonomic classification, for which an expanded comparative analysis was needed, to build on previous assessments and include more clade exclusion-based analysis. For the methods we analyzed, there is no single method that stands out as superior to all others, as there are a wide variety of characteristics in which the methods differ—characteristics that may make them more suitable for certain research group infrastructure, and research projects, than others. Few researchers will have the time to evaluate methods robustly themselves, so may just use the method which is most popular or easiest to use, which would not necessarily be well suited for their particular computational resources and/or goals. This evaluation explains some of the issues researchers should consider when choosing an analysis approach for their metagenomics project, and reveals that very misleading results can occur, in particular notable overprediction of the number of taxa and/or missed taxa, if an inaccurate or unsuitable analysis approach is used. The results from this evaluation will hopefully help guide researchers’ decisions in selecting appropriate analysis methods suitable for their metagenomics studies. As new methods are developed, further evaluations will need to be performed, including with a reference dataset like MetaSimHC, and/or the CAMI approach. This study provides a model for such analyses to compare method accuracies and benefits, and highlights criteria that should be evaluated. It would be very helpful for evaluation purposes if method developers would allow their method’s reference databases to be manipulated, to permit analyses like clade exclusion, to avoid biases that can occur when no clade exclusion is performed (including with unpublished genomes as planned for CAMI, depending on the relatedness of other taxa to these unpublished genomes). Regardless, researchers are strongly encouraged to include appropriate negative and positive controls for their metagenomic experiments, including appropriate *in vitro* mock communities reflecting their expected type of data (high/low diversity, well characterized previously or not, etc.) to help fine tune their methodology as appropriate for their specific experiment. Robust metagenomic data analysis is absolutely critical at this stage of the development of microbiome research as a key research area. Microbiome research promises to be widely applicable to many, studying human health, the environment, agrifood, mining and other natural resource management, but it will only be valuable if high-quality, careful analysis is performed.

## Availability of supporting data

The data sets supporting the results of this article are available in the MG-RAST repository (the *in silico* and *in vitro* test data sets) and accession numbers can be found in Additional file 1: Table S2.

## Additional files

Additional file 1: Supplementary Tables.
**Table S1.** Number of genomes left in the reference databases and training sets of the methods used in the evaluation scenarios. **Table S2.** Datasets used in the evaluation scenarios and their accession numbers. **Table S3.** Number of reads simulated for each organism in the *in silico* datasets. **Table S4.** Methods that were the focus of this evaluation and their version numbers. Methods were run with default parameters except for what we called filtered Kraken which used the kraken-filter script with a threshold score of 0.20. **Table S5.** Number of correctly and incorrectly predicted species^a^ for different thresholds^b^ without clade exclusion, illustrating how some methods vastly overpredict the number species, even when the true number of species is low (in this case the true number of species is 11). **Table S6.** Number of incorrectly predicted species^a^ for different abundance thresholds^b^ with genus clade exclusion. **Table S7.** Number of incorrectly predicted species^a^ for different abundance thresholds^b^ with genus clade exclusion. Even more incorrectly predicted species are predicted under these conditions versus without clade exclusion. (DOCX 34 kb) Additional file 2: Supplementary Figures.
**Figure S1.** Sensitivity and precision with no clade exclusion. Performance of methods on the MetaSimHC dataset of simulated 250 bp reads. **Figure S2.** Taxonomic distance of methods on the MetaSimHC dataset of simulated 250 bp reads with no clade exclusion. **Figure S3.** Taxonomic distance of methods on the MetaSimHC dataset of simulated 250 bp reads with various level of clade exclusion. **Figure S4.** Distributions of misassigned (A) and correct/overpredicted assignments (B) to each taxonomic rank on the MetaSimHC dataset of simulated 250 bp reads under genus clade exclusion. **Figure S5.** Performance as read length is varied. Sensitivity (A), precision (B), and taxonomic distance (C) of methods on the MetaSimHC dataset simulated at lengths of 100, 250, 500, and 1000 bases with genera clade exclusion. **Figure S6.** Performance of FW *in silico* versus FW *in vitro* without clade exclusion. Sensitivity (A) and precision (B) of methods on the FW dataset comparing the performance on the in silico community versus the *in vitro* community. **Figure S7.** Comparison of running time. Running time for the various methods was calculated on a MetaSimHC dataset of 22,000 simulated reads of various read lengths (A), or 22,000 and 44,000 reads of 250 bp (B). (PPTX 100 kb) Additional file 3:
**The proportion of reads assigned at each taxonomic rank on all** ***in silico*** **datasets.** (TXT 213 kb) Additional file 4:
**The numbers of reads misassigned and correctly assigned (or overpredicted) for each rank on all** ***in silico*** **datasets.** (TXT 210 kb) Additional file 5:
**The performance for each of the component genomes on all** ***in silico*** **datasets.** (TXT 674 kb)
