# Supplementary material for: Evaluation of shotgun metagenomics sequence classification methods using in silico and in vitro simulated communities
Source: BMC Bioinformatics. 2015 Nov 4;16:363. doi: 10.1186/s12859-015-0788-5 (PMC4634789; doi:10.1186/s12859-015-0788-5)
Supplement: Additional file 1: Supplementary Tables. — Table S1. Number of genomes left in the reference databases and training sets of the methods used in the evaluation scenarios. Table S2. Datasets used in the evaluation scenarios and their accession numbers. Table S3. Number of reads simulated for each organism in the in silico datasets. Table S4. Methods that were the focus of this evaluation and their version numbers. Methods were run with default parameters except for what we called filtered Kraken which used the kraken-filter script with a threshold score of 0.20. Table S5. Number of correctly and incorrectly predicted speciesa for different thresholdsb without clade exclusion, illustrating how some methods vastly overpredict the number species, even when the true number of species is low (in this case the true number of species is 11). Table S6. Number of incorrectly predicted speciesa for different abundance thresholdsb with genus clade exclusion. Table S7. Number of incorrectly predicted speciesa for different abundance thresholdsb with genus clade exclusion. Even more incorrectly predicted species are predicted under these conditions versus without clade exclusion. (DOCX 34 kb) [file 12859_2015_788_MOESM1_ESM.docx]

**Additional file 1**

**Table S1.** Number of genomes left in the reference databases and training sets of the methods used in the evaluation scenarios

|  | Number of genomes | |
| --- | --- | --- |
| Rank of clade exclusion^a^ | MetaSimHC | Freshwater (FW) |
| None | 2499 | 2499 |
| Species | 2460 | 2388 |
| Genus | 2344 | 2261 |
| Family | 2198 | 2047 |
| Order | 1688 | 1695 |
| Class | 555 | 975 |

^a^Clade exclusion involves removing all sequences from a database at a certain taxonomic level. For example, if performing species-level exclusion for a particular organism, removing all of the genomes from the database of that species.

**Table S2.** Datasets used in the evaluation scenarios and their accession numbers

| Dataset | Read length (bp) | MG-RAST accession number |
| --- | --- | --- |
| MetaSimHC | 100 | 4545484.3 |
| MetaSimHC | 250 | 4548386.3 |
| MetaSimHC | 500 | 4548993.3 |
| MetaSimHC | 1000 | 4548992.3 |
| FW *in silico* | 100 | 4545483.3 |
| FW *in silico* | 250 | 4548385.3 |
| FW *in silico* | 500 | 4548991.3 |
| FW *in silico* | 1000 | 4548990.3 |
| FW *in vitro* | Average 223 | 4545485.3 |

**Table S3.** Number of reads simulated for each organism in the *in silico* datasets

|  | 100 bp | 250 bp | 500 bp | 1000 bp |
| --- | --- | --- | --- | --- |
| Organism | Number of reads | | | |
| MetaSimHC | | | | |
| Agrobacterium tumefaciens str. C58 | 56636 | 22512 | 11318 | 5720 |
| Anabaena variabilis ATCC 29413 | 71330 | 27722 | 13938 | 7298 |
| Archaeoglobus fulgidus DSM 4304 | 21978 | 8550 | 4180 | 2156 |
| Bdellovibrio bacteriovorus HD100 | 37468 | 15032 | 7644 | 3804 |
| Campylobacter jejuni subsp. jejuni 81-176 | 17194 | 6800 | 3414 | 1810 |
| Clostridium acetobutylicum ATCC 824 | 41214 | 16942 | 8118 | 4092 |
| Lactococcus lactis subsp. cremoris SK11 | 26088 | 10514 | 5134 | 2518 |
| Nitrosomonas europaea ATCC 19718 | 27860 | 11316 | 5524 | 2704 |
| Pseudomonas aeruginosa PA7 | 66030 | 26230 | 13688 | 6624 |
| Streptomyces coelicolor A3(2) | 90092 | 36814 | 18198 | 8838 |
| Sulfolobus tokodaii str. 7 | 26830 | 10656 | 5388 | 2708 |
| Total | 482720 | 193088 | 96544 | 48272 |
| FW *in silico* | | | | |
| Bacillus amyloliquefaciens FZB42 | 39074 | 15954 | 7956 | 3856 |
| Bacillus cereus ATCC 14579 | 54922 | 21572 | 10928 | 5564 |
| Burkholderia cenocepacia J2315 | 80712 | 32054 | 15946 | 8244 |
| Escherichia coli str. K-12 substr. MG1655 | 45768 | 18476 | 9216 | 4540 |
| Frankia sp. CcI3 | 54242 | 21816 | 10636 | 5298 |
| Micrococcus luteus NCTC 2665 | 25250 | 9750 | 5072 | 2472 |
| Pseudomonas aeruginosa PAO1 | 62368 | 25302 | 12290 | 6214 |
| Pseudomonas aeruginosa UCBPP-PA14 | 64830 | 26024 | 13304 | 6750 |
| Pseudomonas fluorescens Pf-5 | 71150 | 28546 | 14130 | 7064 |
| Pseudomonas putida KT2440 | 62072 | 24596 | 12224 | 6280 |
| Rhodobacter capsulatus SB 1003 | 38642 | 15698 | 7892 | 3838 |
| Streptomyces coelicolor A3(2) | 90560 | 35870 | 18292 | 8896 |
| Total | 650516 | 259704 | 129930 | 65160 |

**Table S4.** Methods that were the focus of this evaluation and their version numbers. Methods were run with default parameters except for what we called filtered Kraken which used the kraken-filter script with a threshold score of 0.20

| Method | Version |
| --- | --- |
| CARMA3 | 3.0 |
| CLARK | 1.1.3 |
| DiScRIBinATE | 1.0 |
| Kraken | 0.10.2 |
| MEGAN4 | 4.70.4 |
| MetaBin | 1.0 |
| MetaCV | 2.3.0 |
| MetaPhyler | 1.25 |
| PhymmBL | 4.0 |
| RITA | 1.0.1 |
| TACOA | 1.0 |
| MG-RAST | 3.3.7.3 |

**Table S5.** Number of correctly and incorrectly predicted species^a^ for different thresholds^b^ without clade exclusion, illustrating how some methods vastly overpredict the number species, even when the true number of species is low (in this case the true number of species is 11).

|  | No cutoff^b^ | | Cutoff > 0.01%^b^ | | Cutoff > 0.1%^b^ | | Cutoff > 1%^b^ | |
| --- | --- | --- | --- | --- | --- | --- | --- | --- |
| Method | Correct | Incorrect | Correct | Incorrect | Correct | Incorrect | Correct | Incorrect |
| CARMA3 | 11 | 32 | 11 | 2 | 11 | 0 | 11 | 0 |
| CLARK | 11 | 32 | 11 | 9 | 11 | 2 | 11 | 0 |
| DiScRIBinATE RAPSearch2^c^ | N/A | N/A | N/A | N/A | N/A | N/A | N/A | N/A |
| Kraken | 11 | 0 | 11 | 0 | 11 | 0 | 11 | 0 |
| Filtered Kraken | 11 | 0 | 11 | 0 | 11 | 0 | 11 | 0 |
| MEGAN4 BlastN | 11 | 0 | 11 | 0 | 11 | 0 | 11 | 0 |
| MEGAN4 RAPSearch2 | 11 | 63 | 11 | 19 | 11 | 1 | 11 | 0 |
| MetaBin | 11 | 262 | 11 | 36 | 11 | 2 | 11 | 0 |
| MetaCV | 11 | 1166 | 11 | 38 | 11 | 1 | 11 | 0 |
| MetaPhyler | 11 | 7 | 11 | 7 | 11 | 4 | 9 | 1 |
| PhymmBL^c^ | N/A | N/A | N/A | N/A | N/A | N/A | N/A | N/A |
| RITA | 11 | 38 | 11 | 0 | 11 | 0 | 10 | 0 |
| TACOA^c^ | N/A | N/A | N/A | N/A | N/A | N/A | N/A | N/A |
| MG-RAST best hit | 11 | 622 | 11 | 60 | 11 | 6 | 11 | 2 |
| MG-RAST LCA | 11 | 125 | 11 | 7 | 11 | 1 | 11 | 0 |

^a^Using the MetaSimHC dataset of simulated 250 bp reads from 11 species.

^b^A cutoff of > x%, for example 0.01%, would indicate that only species with a predicted abundance of at least x% of the total set of predictions were considered. Correctly predicted species are any of the 11 species that were used to simulate the reads in the dataset, whereas any other predicted species was incorrect.

^c^These methods do not predict to the species level at this read length (they require longer read lengths). See additional analyses at other levels of clade exclusion.

**Table S6.** Number of incorrectly predicted species^a^ for different abundance thresholds^b^ with genus clade exclusion.

| Method | No cutoff^b^ | Cutoff > 0.01%^b^ | Cutoff > 0.1%^b^ | Cutoff > 1%^b^ |
| --- | --- | --- | --- | --- |
| CARMA3 | 71 | 11 | 1 | 1 |
| CLARK | 839 | 467 | 94 | 6 |
| DiScRIBinATE RAPSearch2^c^ | N/A | N/A | N/A | N/A |
| Kraken | 860 | 445 | 95 | 7 |
| Filtered Kraken | 50 | 39 | 13 | 1 |
| MEGAN4 BlastN | 640 | 493 | 79 | 6 |
| MEGAN4 RAPSearch2 | 648 | 354 | 31 | 6 |
| MetaBin | 973 | 320 | 31 | 6 |
| MetaCV | 1263 | 1076 | 84 | 7 |
| MetaPhyler | 9 | 9 | 9 | 1 |
| PhymmBL^c^ | N/A | N/A | N/A | N/A |
| RITA | 934 | 263 | 39 | 14 |
| TACOA^c^ | N/A | N/A | N/A | N/A |
| MG-RAST best hit^d^ | N/A | N/A | N/A | N/A |
| MG-RAST LCA^d^ | N/A | N/A | N/A | N/A |

^a^Using the MetaSimHC dataset of simulated 250 bp reads.

^b^A cutoff of > x%, for example 0.01%, would indicate that only species with a predicted abundance of at least x% of the total set of predictions were considered. Due to genus clade exclusion, it is impossible to correctly predict any of the species, so only incorrect predictions are shown.

^c^These methods do not predict to the species level at this read length (they require longer read lengths). See additional analyses at other levels of clade exclusion.

^d^Could not perform clade-exclusion on MG-RAST

**Table S7.** Number of incorrectly predicted species^a^ for different abundance thresholds^b^ with genus clade exclusion. Even more incorrectly predicted species are predicted under these conditions versus without clade exclusion.

| Method | No cutoff^b^ | Cutoff > 0.01%^b^ | Cutoff > 0.1%^b^ | Cutoff > 1%^b^ |
| --- | --- | --- | --- | --- |
| CARMA3 | 102 | 9 | 4 | 0 |
| DiScRIBinATE RAPSearch2^c^ | N/A | N/A | N/A | N/A |
| Kraken | 741 | 422 | 145 | 10 |
| Filtered Kraken | 87 | 39 | 10 | 5 |
| MEGAN4 BlastN | 447 | 231 | 25 | 2 |
| MEGAN4 RAPSearch2 | 517 | 273 | 32 | 3 |
| MetaBin | 905 | 316 | 36 | 3 |
| MetaCV | 1253 | 901 | 144 | 3 |
| MetaPhyler | 6 | 6 | 4 | 1 |
| PhymmBL^c^ | N/A | N/A | N/A | N/A |
| RITA | 865 | 502 | 182 | 16 |
| TACOA^c^ | N/A | N/A | N/A | N/A |
| MG-RAST best hit^d^ | N/A | N/A | N/A | N/A |
| MG-RAST LCA^d^ | N/A | N/A | N/A | N/A |

^a^Using the FW *in vitro* dataset of sequenced reads from 11 species.

^b^A cutoff of > x%, for example 0.01%, would indicate that only species with a predicted abundance of at least x% of the total set of predictions were considered. Due to genus clade exclusion, it is impossible to correctly predict any of the species, so only incorrect predictions are shown.

^c^These methods do not predict to the species level at this read length (they require longer read lengths). See additional analyses at other levels of clade exclusion.

^d^Could not perform clade exclusion on MG-RAST
