# Supplementary material for: Evaluation of shotgun metagenomics sequence classification methods using in silico and in vitro simulated communities
Source: BMC Bioinformatics. 2015 Nov 4;16:363. doi: 10.1186/s12859-015-0788-5 (PMC4634789; doi:10.1186/s12859-015-0788-5)
Supplement: Additional file 2: Supplementary Figures. — Figure S1. Sensitivity and precision with no clade exclusion. Performance of methods on the MetaSimHC dataset of simulated 250 bp reads. Figure S2. Taxonomic distance of methods on the MetaSimHC dataset of simulated 250 bp reads with no clade exclusion. Figure S3. Taxonomic distance of methods on the MetaSimHC dataset of simulated 250 bp reads with various level of clade exclusion. Figure S4. Distributions of misassigned (A) and correct/overpredicted assignments (B) to each taxonomic rank on the MetaSimHC dataset of simulated 250 bp reads under genus clade exclusion. Figure S5. Performance as read length is varied. Sensitivity (A), precision (B), and taxonomic distance (C) of methods on the MetaSimHC dataset simulated at lengths of 100, 250, 500, and 1000 bases with genera clade exclusion. Figure S6. Performance of FW in silico versus FW in vitro without clade exclusion. Sensitivity (A) and precision (B) of methods on the FW dataset comparing the performance on the in silico community versus the in vitro community. Figure S7. Comparison of running time. Running time for the various methods was calculated on a MetaSimHC dataset of 22,000 simulated reads of various read lengths (A), or 22,000 and 44,000 reads of 250 bp (B). (PPTX 100 kb) [file 12859_2015_788_MOESM2_ESM.pptx]

## Slide 1
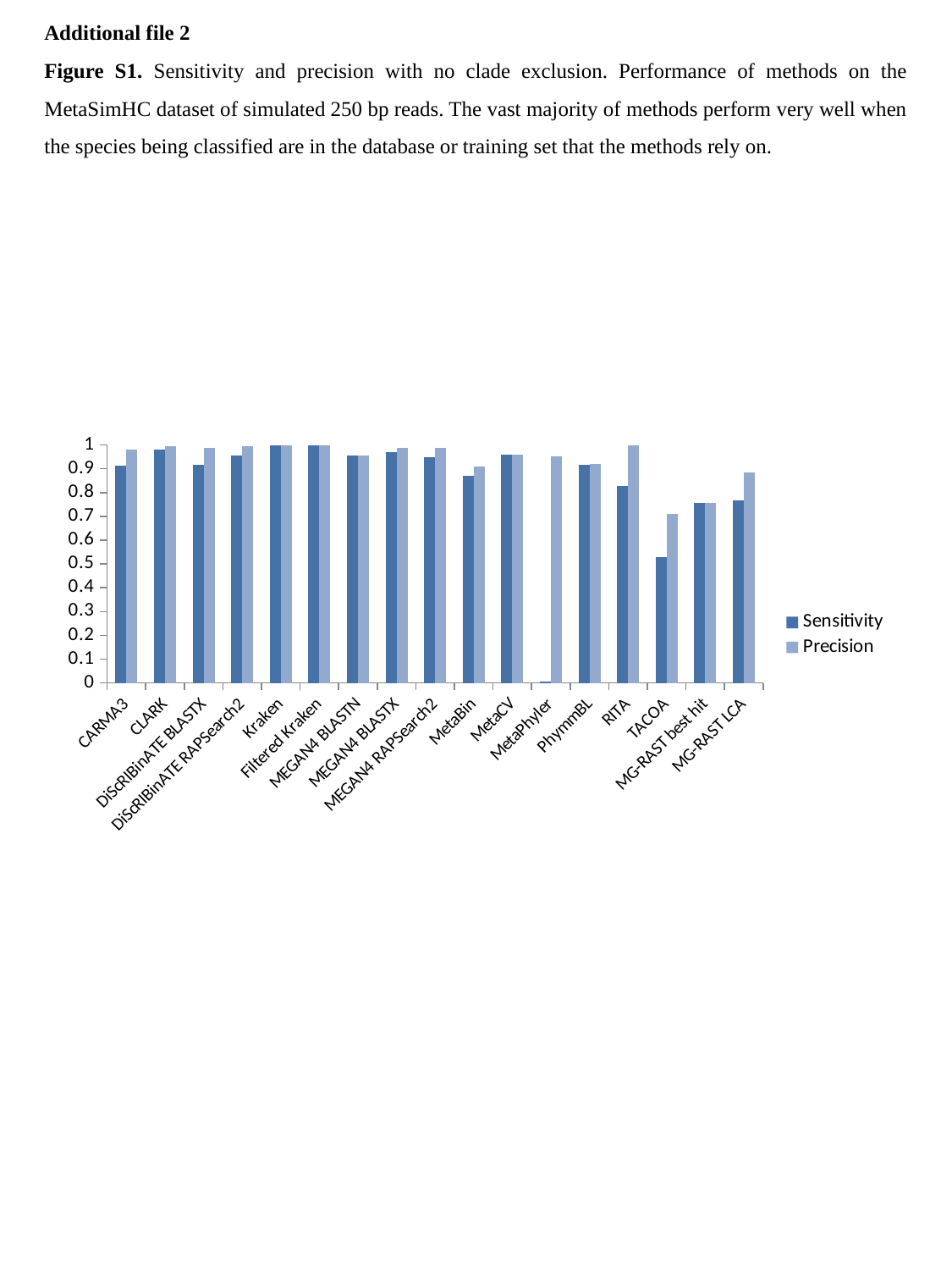

Additional file 2
Figure S1. Sensitivity and precision with no clade exclusion. Performance of methods on the MetaSimHC dataset of simulated 250 bp reads. The vast majority of methods perform very well when the species being classified are in the database or training set that the methods rely on.
### Chart
| Category | Sensitivity | Precision |
|---|---|---|
| CARMA3 | 0.912064612744915 | 0.980915138691824 |
| CLARK | 0.9792540763628411 | 0.994899080955103 |
| DiScRIBinATE BLASTX | 0.9149709069583761 | 0.9884233622724898 |
| DiScRIBinATE RAPSearch2 | 0.9549708644300331 | 0.994622820397899 |
| Kraken | 1.0 | 1.0 |
| Filtered Kraken | 1.0 | 1.0 |
| MEGAN4 BLASTN | 0.956112295664534 | 0.956112295664534 |
| MEGAN4 BLASTX | 0.9698676578338941 | 0.986071007317358 |
| MEGAN4 RAPSearch2 | 0.9485178301415791 | 0.98725771339751 |
| MetaBin | 0.8709597180074841 | 0.9097323407542849 |
| MetaCV | 0.9582216016536882 | 0.959459464256133 |
| MetaPhyler | 0.005762425823622061 | 0.9518458862186712 |
| PhymmBL | 0.91737488506412 | 0.9186208003994942 |
| RITA | 0.8264971738325689 | 0.9984681714609118 |
| TACOA | 0.5302411147372169 | 0.709247174547841 |
| MG-RAST best hit | 0.758261719755056 | 0.758261719755056 |
| MG-RAST LCA | 0.7683387885316539 | 0.882893444818044 |

## Slide 2
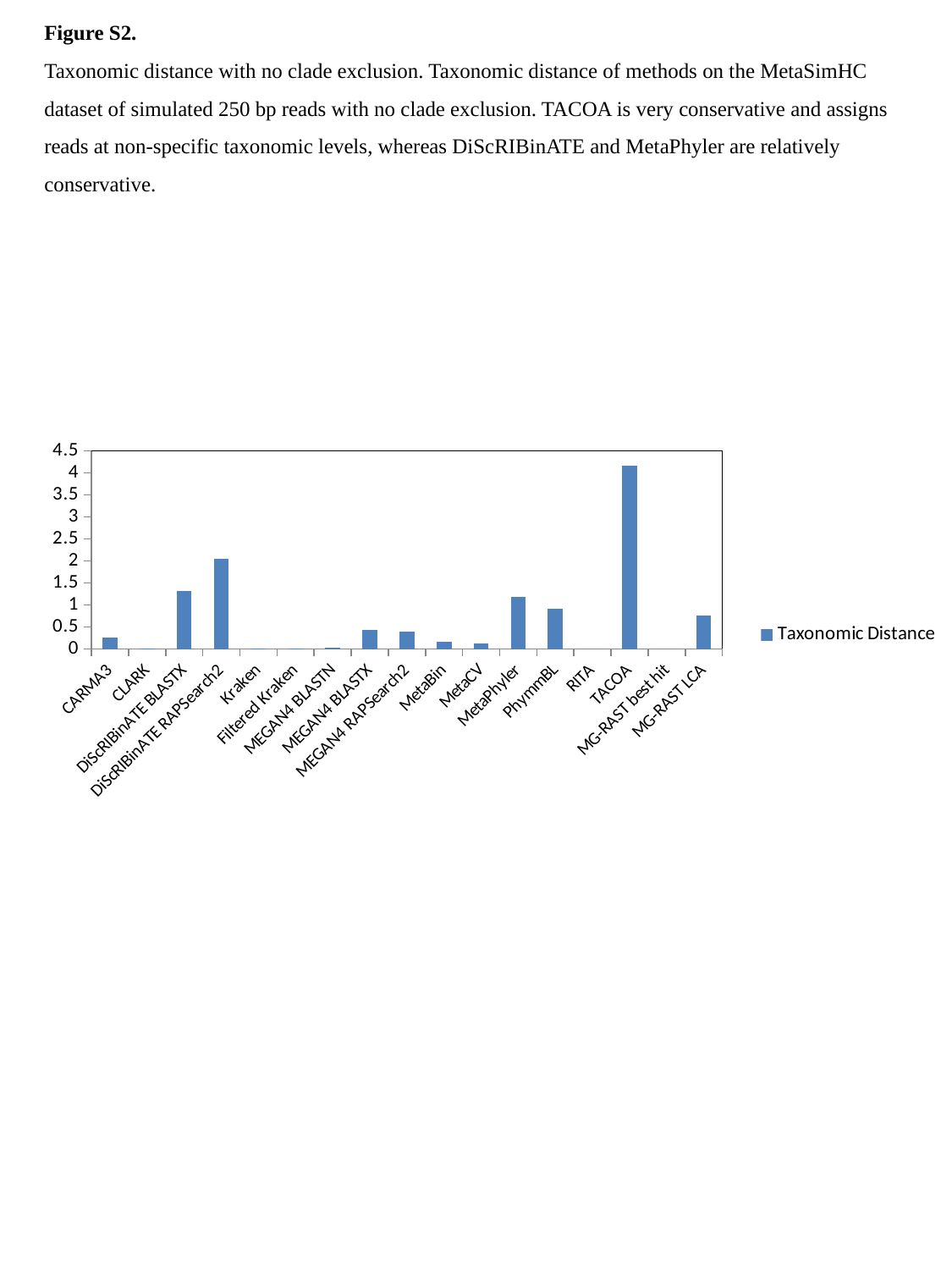

Figure S2.
Taxonomic distance with no clade exclusion. Taxonomic distance of methods on the MetaSimHC dataset of simulated 250 bp reads with no clade exclusion. TACOA is very conservative and assigns reads at non-specific taxonomic levels, whereas DiScRIBinATE and MetaPhyler are relatively conservative.
### Chart
| Category | Taxonomic Distance |
|---|---|
| CARMA3 | 0.26379418053358794 |
| CLARK | 0.006148248085085082 |
| DiScRIBinATE BLASTX | 1.31781078397341 |
| DiScRIBinATE RAPSearch2 | 2.05299004944963 |
| Kraken | 0.007636056121843951 |
| Filtered Kraken | 0.016963156823639504 |
| MEGAN4 BLASTN | 0.0382436692388941 |
| MEGAN4 BLASTX | 0.4425286191649751 |
| MEGAN4 RAPSearch2 | 0.402721319598576 |
| MetaBin | 0.15517758353679603 |
| MetaCV | 0.13214010120466999 |
| MetaPhyler | 1.1879272103538598 |
| PhymmBL | 0.9186208003994942 |
| RITA | 0.0 |
| TACOA | 4.160645771471939 |
| MG-RAST best hit | 0.0 |
| MG-RAST LCA | 0.7563379999999998 |

## Slide 3
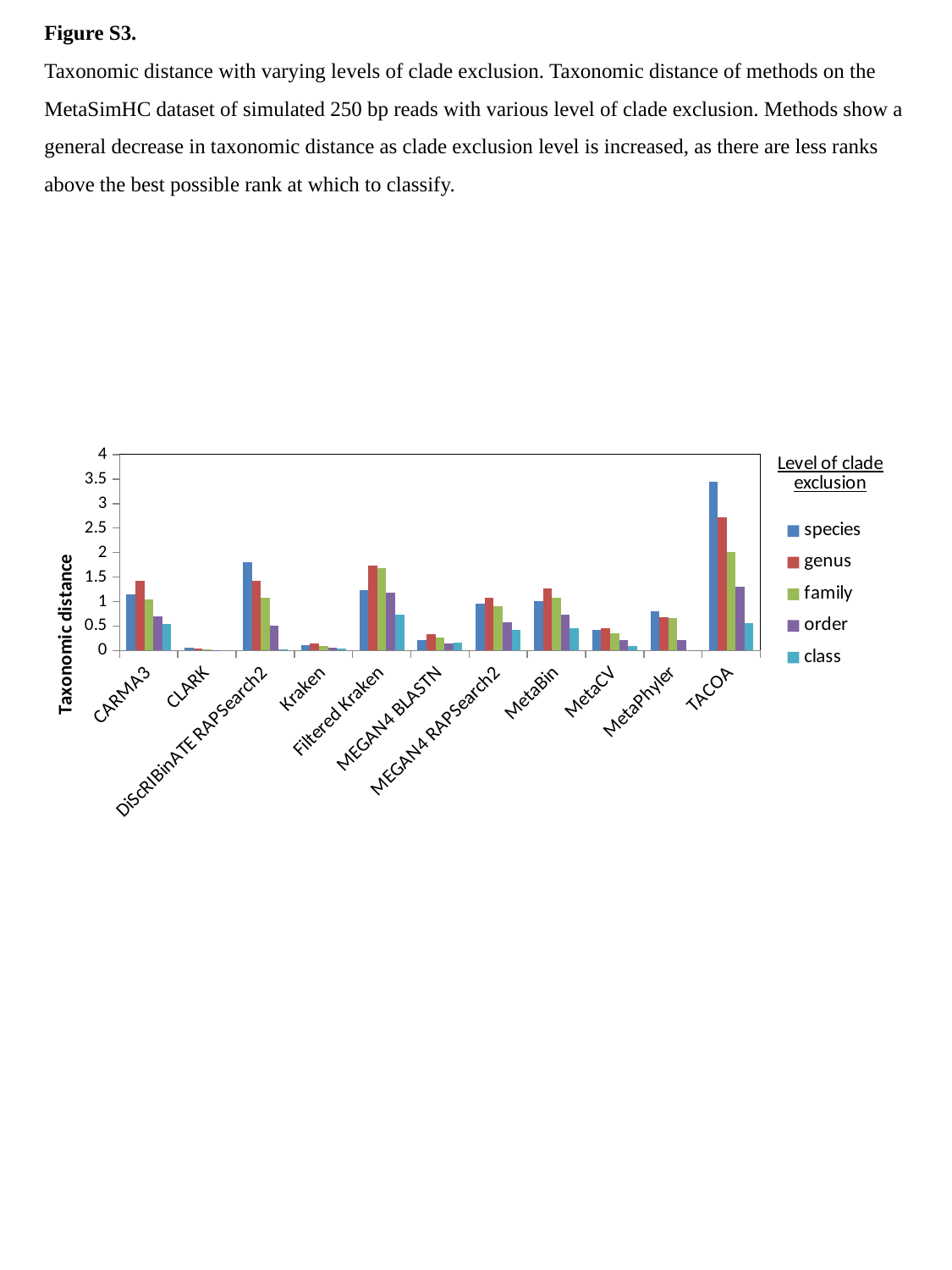

Figure S3.
Taxonomic distance with varying levels of clade exclusion. Taxonomic distance of methods on the MetaSimHC dataset of simulated 250 bp reads with various level of clade exclusion. Methods show a general decrease in taxonomic distance as clade exclusion level is increased, as there are less ranks above the best possible rank at which to classify.
### Chart
| Category | species | genus | family | order | class |
|---|---|---|---|---|---|
| CARMA3 | 1.1535200573656195 | 1.41607890527278 | 1.04119296836788 | 0.7039514378935966 | 0.5461374185298282 |
| CLARK | 0.054610877892078115 | 0.040809119379728996 | 0.021587282724842408 | 0.0011770890070462105 | 0.0 |
| DiScRIBinATE RAPSearch2 | 1.8083202333921695 | 1.4273944433903594 | 1.0782646737984198 | 0.508743824057729 | 0.0297499240117073 |
| Kraken | 0.11006294915227302 | 0.150633294905538 | 0.09920140953728254 | 0.05984334199189323 | 0.043792402443523136 |
| Filtered Kraken | 1.23917276590761 | 1.7295344164830695 | 1.68487963997735 | 1.1863577322360606 | 0.724353166213631 |
| MEGAN4 BLASTN | 0.21615263678378097 | 0.3298989886304192 | 0.2618811643333081 | 0.15107920313194606 | 0.15696874733895005 |
| MEGAN4 RAPSearch2 | 0.953696848257243 | 1.07620384082752 | 0.9031218777275892 | 0.5806501381407658 | 0.4259610201229021 |
| MetaBin | 1.0059789978424494 | 1.27277953373257 | 1.07564153844864 | 0.723668098828238 | 0.4630141805387501 |
| MetaCV | 0.4258460170131621 | 0.448527373473175 | 0.3524152212078483 | 0.21797091281215905 | 0.0861630580452442 |
| MetaPhyler | 0.8017729899470225 | 0.687118922269916 | 0.65933854510168 | 0.21850347389616911 | 0.0 |
| TACOA | 3.44274414872869 | 2.71325060812448 | 2.01215207041251 | 1.29442807422258 | 0.5549176509729988 |

## Slide 4
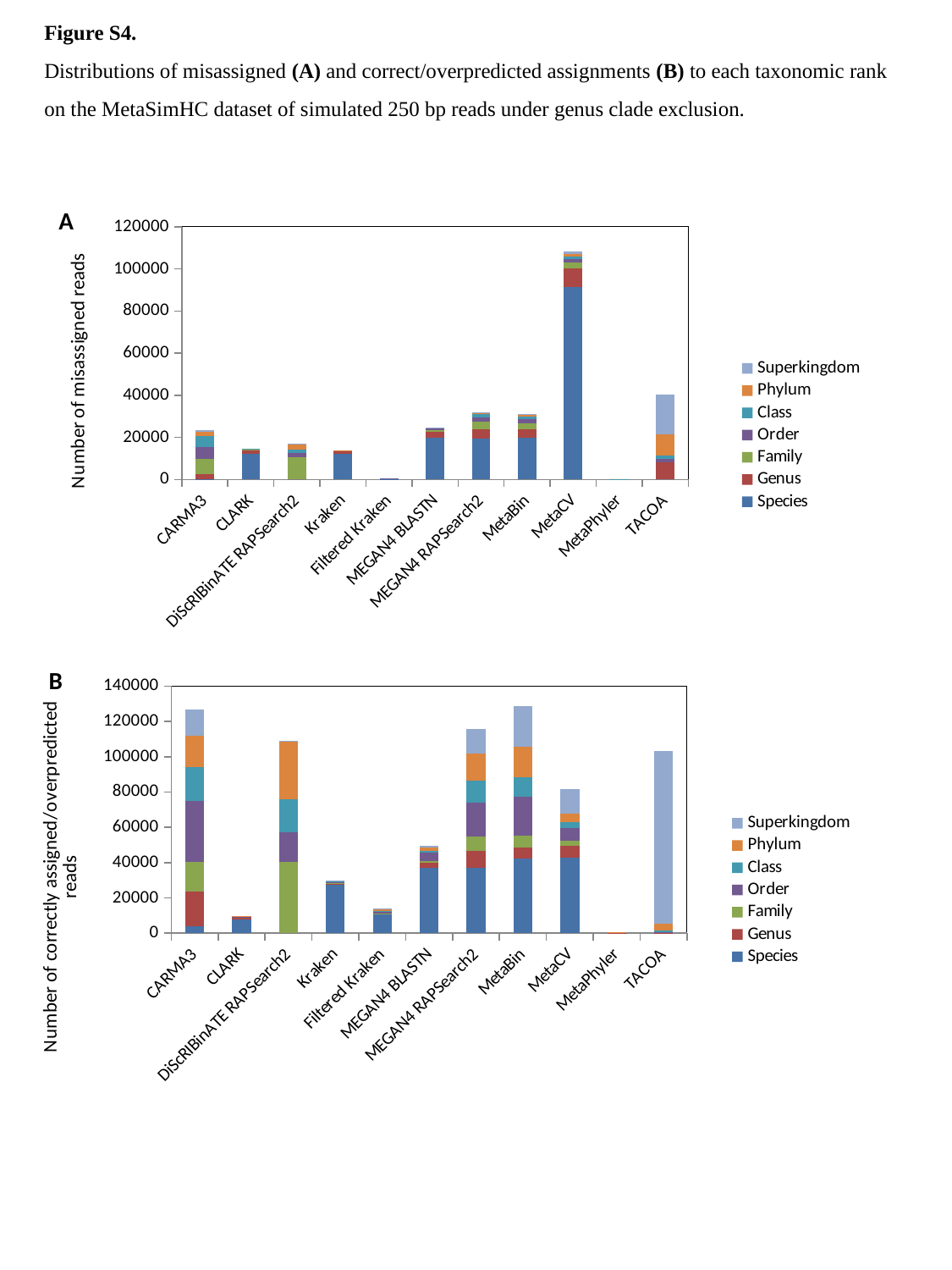

Figure S4.
Distributions of misassigned (A) and correct/overpredicted assignments (B) to each taxonomic rank on the MetaSimHC dataset of simulated 250 bp reads under genus clade exclusion.
### Chart
| Category | Species | Genus | Family | Order | Class | Phylum | Superkingdom |
|---|---|---|---|---|---|---|---|
| CARMA3 | 153.0 | 2519.0 | 7279.0 | 5472.0 | 5012.0 | 2278.0 | 718.0 |
| CLARK | 12321.0 | 1507.0 | 445.0 | 194.0 | 69.0 | 9.0 | 0.0 |
| DiScRIBinATE RAPSearch2 | 0.0 | 0.0 | 10558.0 | 2019.0 | 1717.0 | 2694.0 | 8.0 |
| Kraken | 12268.0 | 1081.0 | 261.0 | 113.0 | 87.0 | 28.0 | 2.0 |
| Filtered Kraken | 262.0 | 73.0 | 26.0 | 58.0 | 8.0 | 0.0 | 0.0 |
| MEGAN4 BLASTN | 19996.0 | 2651.0 | 984.0 | 577.0 | 447.0 | 109.0 | 15.0 |
| MEGAN4 RAPSearch2 | 19271.0 | 4661.0 | 3386.0 | 2189.0 | 1483.0 | 485.0 | 423.0 |
| MetaBin | 19703.0 | 4058.0 | 2789.0 | 2140.0 | 1379.0 | 589.0 | 530.0 |
| MetaCV | 91569.0 | 8671.0 | 2795.0 | 1562.0 | 1054.0 | 1331.0 | 1336.0 |
| MetaPhyler | 20.0 | 19.0 | 23.0 | 93.0 | 12.0 | 5.0 | 0.0 |
| TACOA | 0.0 | 8127.0 | 0.0 | 1481.0 | 1831.0 | 10146.0 | 18589.0 |
### Chart
| Category | Species | Genus | Family | Order | Class | Phylum | Superkingdom |
|---|---|---|---|---|---|---|---|
| CARMA3 | 3843.0 | 19699.0 | 16932.0 | 34225.0 | 19610.0 | 17452.0 | 15117.0 |
| CLARK | 7859.0 | 1053.0 | 237.0 | 187.0 | 229.0 | 132.0 | 0.0 |
| DiScRIBinATE RAPSearch2 | 0.0 | 0.0 | 40485.0 | 16661.0 | 18542.0 | 33096.0 | 400.0 |
| Kraken | 27482.0 | 640.0 | 129.0 | 779.0 | 201.0 | 346.0 | 243.0 |
| Filtered Kraken | 10530.0 | 644.0 | 151.0 | 1063.0 | 380.0 | 529.0 | 595.0 |
| MEGAN4 BLASTN | 37082.0 | 2881.0 | 1079.0 | 4641.0 | 1019.0 | 1817.0 | 1196.0 |
| MEGAN4 RAPSearch2 | 37031.0 | 9343.0 | 8176.0 | 19391.0 | 12302.0 | 15597.0 | 13624.0 |
| MetaBin | 42280.0 | 6438.0 | 6702.0 | 21751.0 | 10993.0 | 17446.0 | 23123.0 |
| MetaCV | 42619.0 | 6896.0 | 2957.0 | 6916.0 | 3380.0 | 5162.0 | 13690.0 |
| MetaPhyler | 9.0 | 127.0 | 356.0 | 60.0 | 155.0 | 39.0 | 0.0 |
| TACOA | 0.0 | 160.0 | 0.0 | 512.0 | 701.0 | 4019.0 | 98030.0 |

## Slide 5
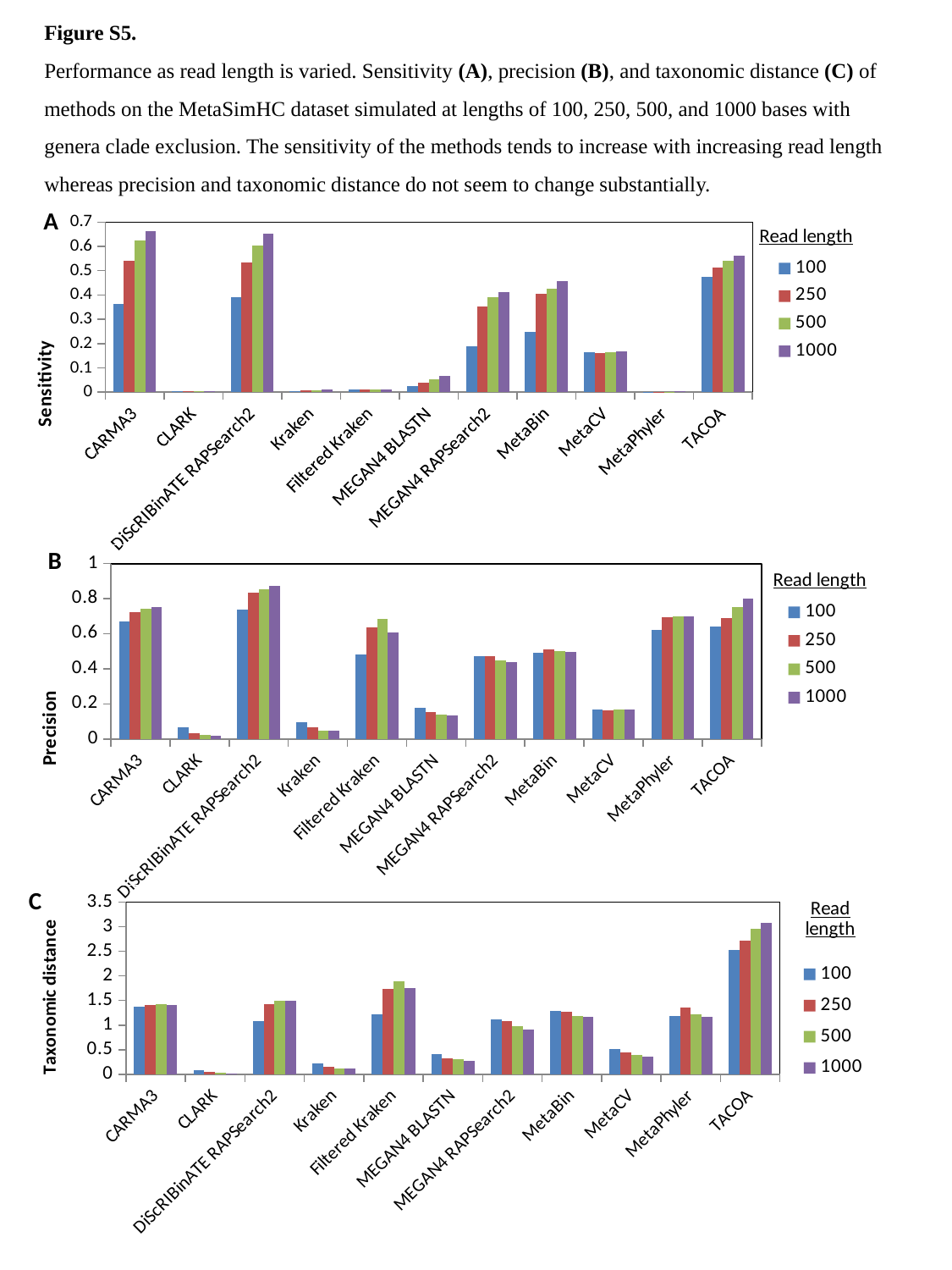

Figure S5.
Performance as read length is varied. Sensitivity (A), precision (B), and taxonomic distance (C) of methods on the MetaSimHC dataset simulated at lengths of 100, 250, 500, and 1000 bases with genera clade exclusion. The sensitivity of the methods tends to increase with increasing read length whereas precision and taxonomic distance do not seem to change substantially.
### Chart
| Category | 100 | 250 | 500 | 1000 |
|---|---|---|---|---|
| CARMA3 | 0.362736319218556 | 0.5408983354570731 | 0.6240087555400742 | 0.6628514376821352 |
| CLARK | 0.003764236464079071 | 0.0033702933619818706 | 0.0036215894236989506 | 0.0037318760840649202 |
| DiScRIBinATE RAPSearch2 | 0.389691793013227 | 0.5338660873696569 | 0.6027616028504231 | 0.6521622123926031 |
| Kraken | 0.005796366926412061 | 0.007243239231428771 | 0.00890786092761184 | 0.011660782590963802 |
| Filtered Kraken | 0.010787770211188805 | 0.012122270330360901 | 0.011990395599851001 | 0.011320376111964701 |
| MEGAN4 BLASTN | 0.025540286407562503 | 0.0392546197313107 | 0.05200132674858371 | 0.0664774950449881 |
| MEGAN4 RAPSearch2 | 0.18948232742760704 | 0.3511365154475601 | 0.390940110105539 | 0.410192179078164 |
| MetaBin | 0.246804258409854 | 0.406134568771587 | 0.42661823287661904 | 0.457517257314372 |
| MetaCV | 0.164696179968426 | 0.161022988985705 | 0.165562998135585 | 0.16815086508844698 |
| MetaPhyler | 0.0007390340020998432 | 0.0014585521741271104 | 0.0021342637757639804 | 0.00311105965102862 |
| TACOA | 0.47426522399401005 | 0.5128327679017969 | 0.5406583126804873 | 0.5609084104617892 |
### Chart
| Category | 100 | 250 | 500 | 1000 |
|---|---|---|---|---|
| CARMA3 | 0.6684274946850071 | 0.7218985468339011 | 0.741495018992957 | 0.7521237389684161 |
| CLARK | 0.0651117112684038 | 0.03254471203241311 | 0.022054020993595 | 0.017402766120695898 |
| DiScRIBinATE RAPSearch2 | 0.7381466255225941 | 0.83388799732505 | 0.8543897451779419 | 0.870315034823326 |
| Kraken | 0.09814401688691178 | 0.0658552383855158 | 0.0491533558689585 | 0.045742824418955 |
| Filtered Kraken | 0.48060609918918507 | 0.6350093202628792 | 0.6858654306714591 | 0.6086826064803441 |
| MEGAN4 BLASTN | 0.180030919885815 | 0.15236234510903504 | 0.137478826583117 | 0.13258066233495497 |
| MEGAN4 RAPSearch2 | 0.473164349876314 | 0.47280489525353603 | 0.4471299631372401 | 0.4372790671647541 |
| MetaBin | 0.48996350106176007 | 0.5098465106818161 | 0.499006254730505 | 0.4940045305884271 |
| MetaCV | 0.169312594496535 | 0.164000341508115 | 0.167682229853373 | 0.169520498322184 |
| MetaPhyler | 0.6218306615887171 | 0.69490199719966 | 0.6986267117795171 | 0.6997877087849039 |
| TACOA | 0.6388290766478734 | 0.6888490041654922 | 0.7516663774848061 | 0.800587382346577 |
### Chart
| Category | 100 | 250 | 500 | 1000 |
|---|---|---|---|---|
| CARMA3 | 1.3769398510776598 | 1.41607890527278 | 1.4258020752514697 | 1.40360599124354 |
| CLARK | 0.08087341050435051 | 0.040809119379728996 | 0.028408901783908203 | 0.02028236652032671 |
| DiScRIBinATE RAPSearch2 | 1.0754153812043599 | 1.4273944433903596 | 1.4864361321326798 | 1.4973703708245598 |
| Kraken | 0.21344041208113906 | 0.150633294905538 | 0.11770707383157501 | 0.113821834806783 |
| Filtered Kraken | 1.2167307434783998 | 1.7295344164830697 | 1.8882586995578001 | 1.7496846590132198 |
| MEGAN4 BLASTN | 0.41445953877406905 | 0.3298989886304191 | 0.2979284467456551 | 0.2813766448640531 |
| MEGAN4 RAPSearch2 | 1.11258913823246 | 1.07620384082752 | 0.9786048453689331 | 0.9162112355593881 |
| MetaBin | 1.28681874785308 | 1.27553743319316 | 1.1929119558132601 | 1.15991910078608 |
| MetaCV | 0.5140676640794329 | 0.448527373473175 | 0.3992502222721341 | 0.3520622279006641 |
| MetaPhyler | 1.18522348253579 | 1.3543502552845799 | 1.2274753872819597 | 1.1641231201809201 |
| TACOA | 2.52118687965475 | 2.71325060812448 | 2.9517149106400797 | 3.0783348837710705 |

## Slide 6
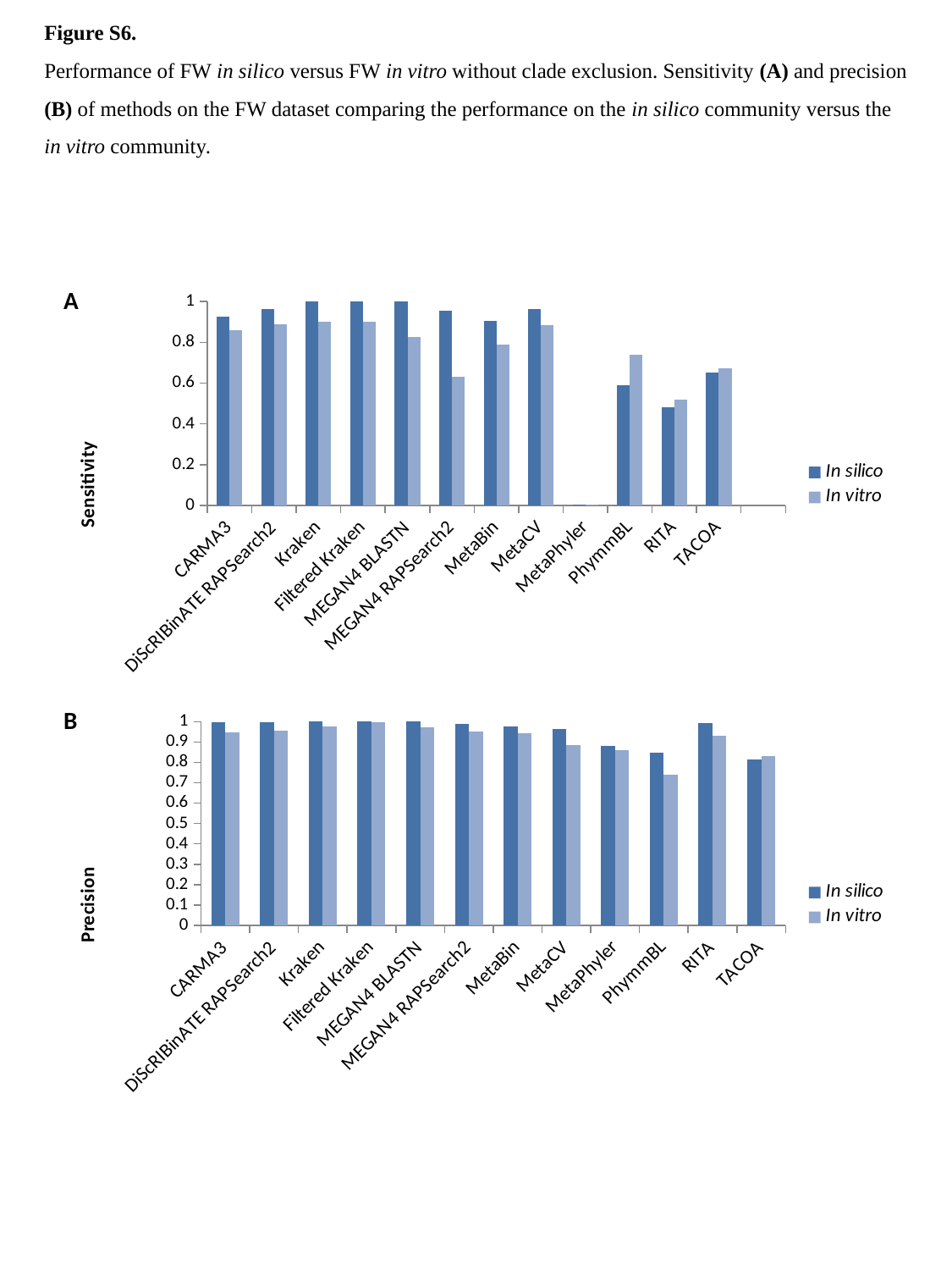

Figure S6.
Performance of FW in silico versus FW in vitro without clade exclusion. Sensitivity (A) and precision (B) of methods on the FW dataset comparing the performance on the in silico community versus the in vitro community.
### Chart
| Category | In silico | In vitro |
|---|---|---|
| CARMA3 | 0.925069018921285 | 0.8581880525901344 |
| DiScRIBinATE RAPSearch2 | 0.96197225776042 | 0.890161445198675 |
| Kraken | 1.0 | 0.9022258106316602 |
| Filtered Kraken | 1.0 | 0.9026178775887223 |
| MEGAN4 BLASTN | 0.9999966332233522 | 0.8243971970535172 |
| MEGAN4 RAPSearch2 | 0.9546192175611075 | 0.6328924241367052 |
| MetaBin | 0.9064911453774158 | 0.7892008811538732 |
| MetaCV | 0.964736381388459 | 0.8837687602377646 |
| MetaPhyler | 0.0038212914955221907 | 0.004638351458123592 |
| PhymmBL | 0.591239647161807 | 0.7381989507225002 |
| RITA | 0.4823715574708771 | 0.520342626649256 |
| TACOA | 0.6508753619284904 | 0.672494509401301 |
### Chart
| Category | In silico | In vitro |
|---|---|---|
| CARMA3 | 0.995128735390733 | 0.946068304689904 |
| DiScRIBinATE RAPSearch2 | 0.9963385929735862 | 0.9534097500738432 |
| Kraken | 1.0 | 0.9762325051320676 |
| Filtered Kraken | 1.0 | 0.9988858778583852 |
| MEGAN4 BLASTN | 1.0 | 0.9732598505501404 |
| MEGAN4 RAPSearch2 | 0.9866173487318486 | 0.952190757079657 |
| MetaBin | 0.977544285138565 | 0.942264131482591 |
| MetaCV | 0.9653246372612763 | 0.8854604286370568 |
| MetaPhyler | 0.8791634391944232 | 0.860135551447936 |
| PhymmBL | 0.848562454699203 | 0.7391715157382732 |
| RITA | 0.991364636525927 | 0.930976471007859 |
| TACOA | 0.8144167902399565 | 0.8303828212502508 |

## Slide 7
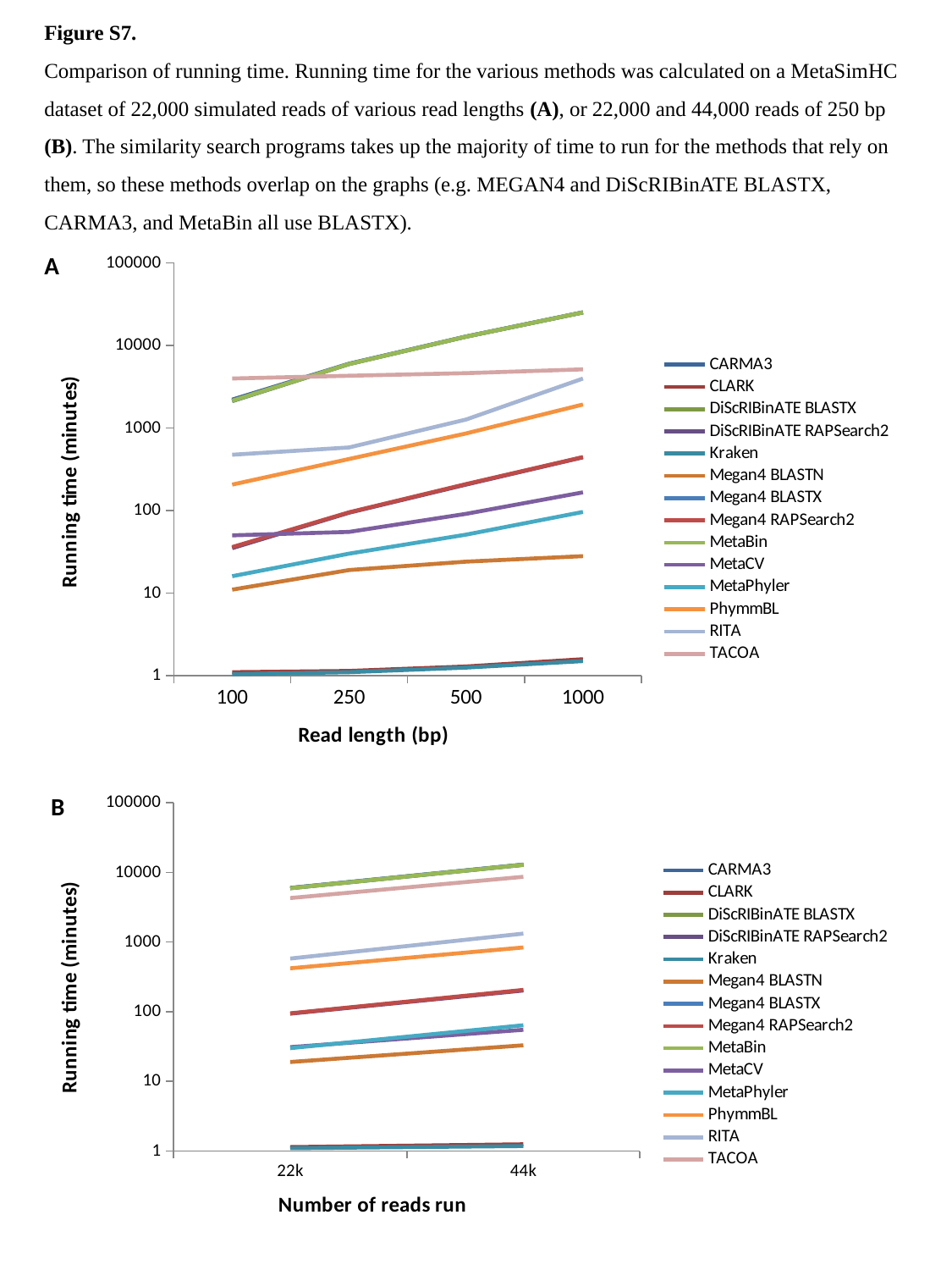

Figure S7.
Comparison of running time. Running time for the various methods was calculated on a MetaSimHC dataset of 22,000 simulated reads of various read lengths (A), or 22,000 and 44,000 reads of 250 bp (B). The similarity search programs takes up the majority of time to run for the methods that rely on them, so these methods overlap on the graphs (e.g. MEGAN4 and DiScRIBinATE BLASTX, CARMA3, and MetaBin all use BLASTX).
### Chart
| Category | CARMA3 | CLARK | DiScRIBinATE BLASTX | DiScRIBinATE RAPSearch2 | Kraken | Megan4 BLASTN | Megan4 BLASTX | Megan4 RAPSearch2 | MetaBin | MetaCV | MetaPhyler | PhymmBL | RITA | TACOA |
|---|---|---|---|---|---|---|---|---|---|---|---|---|---|---|
| 100 | 2201.0 | 1.1 | 2113.0 | 35.0 | 1.04 | 11.0 | 2125.0 | 36.0 | 2136.0 | 50.0 | 16.0 | 206.0 | 474.0 | 3971.0 |
| 250 | 6004.0 | 1.1400000000000001 | 5943.0 | 94.0 | 1.1 | 19.0 | 5950.0 | 95.0 | 5941.0 | 55.0 | 30.0 | 421.0 | 581.0 | 4291.0 |
| 500 | 12790.0 | 1.29 | 12761.000000000002 | 206.0 | 1.25 | 24.0 | 12761.000000000002 | 209.0 | 12761.000000000002 | 91.0 | 51.0 | 858.0 | 1265.0 | 4611.0 |
| 1000 | 25135.0 | 1.58 | 25081.0 | 441.0 | 1.5 | 28.0 | 25081.0 | 444.0 | 25081.0 | 166.0 | 96.0 | 1925.0 | 3965.0 | 5125.0 |
### Chart
| Category | CARMA3 | CLARK | DiScRIBinATE BLASTX | DiScRIBinATE RAPSearch2 | Kraken | Megan4 BLASTN | Megan4 BLASTX | Megan4 RAPSearch2 | MetaBin | MetaCV | MetaPhyler | PhymmBL | RITA | TACOA |
|---|---|---|---|---|---|---|---|---|---|---|---|---|---|---|
| 22k | 6004.0 | 1.1400000000000001 | 5943.0 | 94.0 | 1.1 | 19.0 | 5950.0 | 95.0 | 5941.0 | 31.0 | 30.0 | 421.0 | 581.0 | 4291.0 |
| 44k | 12982.0 | 1.25 | 12855.0 | 202.0 | 1.1800000000000002 | 33.0 | 12864.0 | 206.0 | 12879.0 | 55.0 | 64.0 | 838.0 | 1326.0 | 8658.0 |
